# Enhanced intelligent water drops algorithm for multi-depot vehicle routing problem

**DOI:** 10.1371/journal.pone.0193751

**Published:** 2018-03-19

**Authors:** Absalom E. Ezugwu, Francis Akutsah, Micheal O. Olusanya, Aderemi O. Adewumi

**Affiliations:** 1 School of Mathematics, Statistics and Computer Science, University of Kwazulu-Natal, Durban, South Africa; 2 Department of Computer Science, Federal University Lafia, Lafia, Nasarawa State, Nigeria; Universita degli Studi di Catania, ITALY

## Abstract

The intelligent water drop algorithm is a swarm-based metaheuristic algorithm, inspired by the characteristics of water drops in the river and the environmental changes resulting from the action of the flowing river. Since its appearance as an alternative stochastic optimization method, the algorithm has found applications in solving a wide range of combinatorial and functional optimization problems. This paper presents an improved intelligent water drop algorithm for solving multi-depot vehicle routing problems. A simulated annealing algorithm was introduced into the proposed algorithm as a local search metaheuristic to prevent the intelligent water drop algorithm from getting trapped into local minima and also improve its solution quality. In addition, some of the potential problematic issues associated with using simulated annealing that include high computational runtime and exponential calculation of the probability of acceptance criteria, are investigated. The exponential calculation of the probability of acceptance criteria for the simulated annealing based techniques is computationally expensive. Therefore, in order to maximize the performance of the intelligent water drop algorithm using simulated annealing, a better way of calculating the probability of acceptance criteria is considered. The performance of the proposed hybrid algorithm is evaluated by using 33 standard test problems, with the results obtained compared with the solutions offered by four well-known techniques from the subject literature. Experimental results and statistical tests show that the new method possesses outstanding performance in terms of solution quality and runtime consumed. In addition, the proposed algorithm is suitable for solving large-scale problems.

## Introduction

Managing a fleet of vehicles outsourced for the distribution of a specific number of products to a set of customers with specific supply and demand requirements, is considered an important challenge in dealing with distribution problems. The challenge in this case, is not only restricted to making decisions on the number of vehicles to be dispatched on the road, but in deciding how a customer receives a service and which customer receives services first, based on assigned priority [1]. This type of problem can be presented as a vehicle routing problem (VRP) modeled by using graph theory. The expected performance metrics involve determining the optimal sequence of customers to be visited by each vehicle, which satisfy the criteria such as travel time, the length of route and the cost involved in the operation [2]. The VRP optimization problem is widely-studied with several attractive solutions and different implementation techniques proposed in the literature [3, 4, 5, 6]. Similarly, several variants of the VRP, of which the design concepts are based on the operational mechanism and mathematical modeling of the problem’s diverse conditions in real-world applications, have been scrutinized recently by researchers [7, 8, 9, 10, 11]. Some of these variants of the VRP include capacitated vehicle routing problems (CVRP), heterogeneous fleet vehicle routing problems (HVRP), multi-depot vehicle routing problems (MDVRP), periodic vehicle routing problems (PVRP), stochastic vehicle routing problems (SVRP), and vehicle routing problems with time windows (VRPTW) [12, 11].

The classical VRP is the most studied version of the VRP’s constrained optimization problem, drawing substantial interest in literature. In the classical VRP, there is only one depot and all vehicles start and end their routes in that depot [13, 14]. However, in present day real-world applications the classical VRP has limited practical application due to the complex structural orientation of the real-world problem. The VRP and its variants, including the multi-depot vehicle routing problem (MDVRP) discussed in this paper, are considered to be NP-hard problems, since they cannot be solved in polynomial time [15, 16]. In other words, finding an optimal solution for the VRP usually is very difficult to achieve, requiring an extensive amount of computational effort.

Since VRP is an NP-hard problem, several methods are proposed for solving VRPs and its variants. These methods include the exact algorithms, heuristics and metaheuristics techniques. However, using the exact algorithms are usually not effective, due to their time consuming characteristics when applied, which become obvious when the number of nodes increases and connectivity causes a drastic increase in the computational time required. Examples of the exact solutions are column generation [17, 18], integer programming [19], branch-and-bound and branch-and-cut algorithms and set-covering-based solution methods [11]. As a result of the underperformances of the exact algorithms with large graphs, a large number of classical heuristics or evolutionary computing methods and metaheuristics algorithms, has been proposed to solve the VRPs. This include simulated annealing [20], Tabu search [21], genetic algorithms [22], ant algorithms [23], neural networks [24], particle swarm optimization [25], symbiotic organisms search algorithm [26], and intelligent water drop algorithms [27, 28].

In the MDVRP, more than one depot exists and each customer is visited by one of the vehicles based at one of the several depots. The MDVRP has more practical applications than the classical VRP, as it is inclined toward offering better services to multiple customers from several depots. Although the MDVRP is said to be more robust in its application, it presents a greater challenge to the decision makers in determining which customer is visited by a vehicle from which depot without exceeding the actual capacity constraints of each vehicle. The inherent complexity of the MDVRP makes it difficult to solve the problem on a larger scale. Therefore, the MDVRP can then rather be viewed as a clustering problem, in which case grouping is performed to cluster the customers based on distance between the customers and the depots. Therefore, the problem can be solved in two phases simultaneously. The first phase is the allocation of customers to depots and the second phase is the routing of customers allocated to the same depots through routes. To adequately handle large problems, a reasonable approach would be to further decompose the two phases into the following sub-problems, namely clustering, routing, scheduling and optimization. Although most of the existing classical heuristic algorithms, such as genetic algorithms [22] and simulated annealing [20] guarantee near-optimal solutions, this is not always the case when solving MDVRP with large problem sizes, where for example the number of possible solutions would increase with every slightest increase in the number of decision variables. Consequently, the computational effort is most likely to increase likewise, thereby supporting the view that classical based heuristics are not computationally efficient in solving MDVRPs with large problem sizes. However, several hybrid evolutionary algorithms have proven to perform better, in terms of being able to produce better solution quality than their classical counterparts [15, 29, 30, 31], especially in cases of large sized problems. Therefore, this serves as a motivation for proposing a hybrid metaheuristic algorithm that combines both the intelligent water drop (IWD) algorithm and simulated annealing (SA) local search metaheuristic, denoted here as IWD-SA to solve the MDVRP.

IWD is a graph-based metaheuristic algorithm, which makes it suitable for modeling and solving most VRPs and their variants including the MDVRP. The IWD algorithm is a very simple and effective population-based optimization technique, using a constructive approach in finding optimal solution for a given problem [32, 33]. The IWD is inspired by the natural phenomena, based on the idea of water drops and their interactions with soils in river beds. The process is such that each water drop would construct a solution by traversing in the problem search space, at the same time modifying its environment. IWD has found applications in dealing with a wide range of optimization problems which include the well-known travelling salesman problem [34], VRP and its variants [29] and software quality assurance testing [35]. Results from different researchers’ work, have shown that the IWD algorithm compete favorably well with the other state-of-the-art metaheuristic algorithms [27, 28, 29].

This paper proposes the development of a hybrid metaheuristic algorithm based on the IWD algorithm and the SA local search heuristic. Since the SA algorithm has been applied to solve a number of optimization problems with fairly good results in most cases [36], it was selected for its high and better objective values, and for the SA algorithm’s ability to move from the current solution to the neighborhood solutions. This invariably helps the search process escape from the local minima in its search for the global optima by using the specified acceptance probability criteria to either accept or reject solutions with worse objective values. However, the computation of the acceptance probability function consumes a large number of system resources, more specifically CPU time. This is as a result of the exponential calculation required to determine the probability of acceptance or rejection of a new solution. Therefore, approximating the calculation of this function without compromising the decision rule and solution quality, can significantly improve the performance of the framework in terms of cost of execution. In addition, this paper aims to find an even more efficient and effective search strategy and an optimal set of performance parameters to achieve better results and faster convergence speed, especially with MDVRP benchmark problems with graphs ranging from 50 up to 360 nodes. In addition, the comparative analysis of the hybrid methods proposed in this paper is also novel. Therefore, this can be considered as the primary motivation for developing the proposed hybrid algorithm, which consists of the Graph-based IWD and SA search algorithms. The proposed hybrid algorithm is enhanced with the capability of improved IWD and SA algorithms to explore and exploit the solution search space of the MDVRP in a more efficient and effective way. The proposed algorithm is evaluated on thirty three (33) MDVRP benchmark instances. The experimental results obtained demonstrate the efficiency of this method over other compared algorithms from the existing literature [37, 30, 10].

The main technical contributions of this paper are the

- design and implementation of an improved IWD based SA algorithm for solving MDVRP;
- employment of some new adjustments features to SA probability of acceptance criteria function to improve the overall computation speed of the proposed IWD-SA algorithm;
- comparative analysis of the proposed IWD-SA method presented in this paper, in terms of computational cost incurred by the exponential probability function execution of the SA technique; and
- detailed evaluation and statistical analysis of results obtained by the proposed IWD-SA method against its classical approach (that is the standard IWD) and other existing techniques from literature.

This article is structured as follows: The paper starts with the brief discussion on the basics of MDVRP, problem formulation and related works. This is followed by a discussion of the two metaheuristic algorithms, namely IWD and SA, the presentation of hybrid methods, and their applications to solve MDVRP. Presented next are extensive computational results and discussions on the comparative analysis of the obtained results using statistical methods. Finally, the concluding remarks and future directions for the study are presented.

## Multi-depot vehicle routing problem

**Fig 1:**
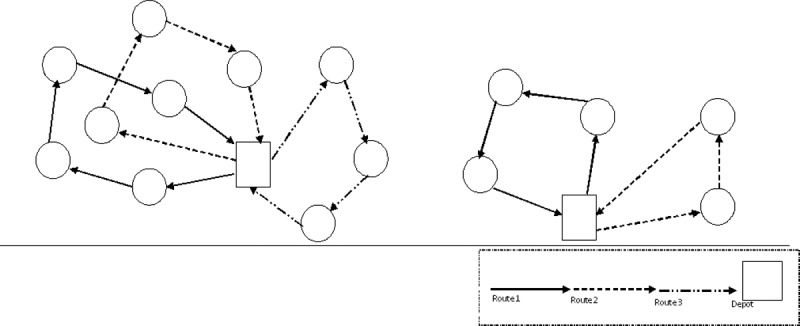
MDVRP model with 2 depots and 15 customers.

The MDVRP was presented as a least cost problem, with the objective of finding routes with the least cost from each designated depot to a set of geographically located customers [36]. In modeling the MDVRP, certain assumptions regarding the vehicle routing plan, capacity, and customers are considered. Firstly, each route begins and ends at the same depot. Secondly, each customer is served exactly once a by vehicle. Thirdly, the total demand on each route is less than, or equal to the capacity of the vehicle assigned to that route. Finally, customers’ demand can be met. An example illustration of the MDVRP with 2 depots and 15 customers is shown in Fig 1. In solving the MDVRP, three decision making processes are involved [38]. The first is clustering, which deals with the grouping of customers to be served by the same depot, based on the distance of each customer from the servicing depots. The second is routing, which is the assignment of customers of the same depot to several routes in such a way that the capacity constraint of the vehicle is not violated. The last is scheduling, which handles the delivery sequence of each route in every depot.

The main objective of the MDVRP is to minimize the total delivery distance or time spent in attending to each customer. As shown in Fig 1, the two rectangular boxes represent the actual depots, while the circles represent the actual customers to be visited. If Fig 1 is defined in terms of a complete undirected graph *G* = (*V*, *E*, *d*), where the set *V* = {1,2,…,*n*} is the node set, *E* = {(*i*,*i*+1): *i*, *i* + 1 ∈ *V*, *i* < *i*+1} is an edge set. A cost matrix *D* = {*d*_*i*,*i*+1_, *v*_*i*_, *v*_*i*+1_ ∈ *V*} corresponding to the distance is defined on *E*. The cost matrix satisfies the triangle inequality whenever *d*_*i*,*k*_ + *d*_*i*+1,*l*_ ≤ *d*_*i*,*l*_ + *d*_*i*+1,*k*_ for all 1 ≤ *i* < *i* + 1 ≤ *n*,1 ≤ *k* < *l* ≤ *n*, or *d*_*i*,*i*+1_ ≤ *d*_*i*,*k*_ + *d*_*k*,*i*+1_, for all *i*, *i* + 1, *k*. In particular, this is the case of planer problems for which the nodes are points *p*_*i*_ = (*x*_*i*_, *y*_*i*_) in the plane, and $d_{i,i+1}=\sqrt{{\left(x_{i}-x_{i+1}\right)}^{2}+{\left(y_{i}-y_{i+1}\right)}^{2}}$ is the Euclidean distance. The triangle inequality is also satisfied if *d*_*i*,*i*+1_ is the length of a shortest path from *i* to *i* + 1 on *G*. For the given MDVRP, the distance between customer *i* and depot *h*, is represented as $d(i,h)=\sqrt{{\left(x_{i}-x_{h}\right)}^{2}+{\left(y_{i}-y_{h}\right)}^{2}}$. The calculated Euclidean distance between customers and depots can then be used to make grouping decision of assigning customers to specific depots and routing decision of assigning customers on the same link to several routes.

According to Ho *et al*. [38], a feasible solution can easily be generated based on three steps namely, clustering, routing, and scheduling. The clustering process is achieved by computing the distance matrix, since the main objective of the MDVRP is to minimize the total time spent in serving each customer. Therefore, customers are clustered according to the nearest depots to them. Considering the example given in Fig 1, with two available depots say *h*_*A*_ and *h*_*B*_, each customer is assigned to exactly one depot based on the following conditions:

If *d*(*c*_*i*_, *h*_*A*_) < *d*(*c*_*i*_, *h*_*B*_), assign *c*_*i*_ to *h*_*A*_

If *d*(*c*_*i*_, *h*_*A*_) > *d*(*c*_*i*_, *h*_*B*_), assign *c*_*i*_ to *h*_*B*_

If *d*(*c*_*i*_, *h*_*A*_) = *d*(*c*_*i*_, *h*_*B*_), assign *c*_*i*_ to either *h*_*A*_ or *h*_*B*_

where $d(c_{i},h_{k})=\sqrt{{\left(x_{c_{i}}-x_{h_{k}}\right)}^{2}+{\left(y_{c_{i}}-y_{h_{k}}\right)}^{2}}$ represents the distance between customer *c*_*i*_ and depot *h*_*k*_.

The scheduling process entails sequencing the assignments of vehicle to customers based on the customers’ proximity to each other, such that no customer is visited more than once. In the proposed method, the parallelism capability of the IWD algorithm is employed to solve the MDVRP using similar steps of clustering and routing techniques described above.

### Related work

Literature reviewed shows that several metaheuristic approaches have been proposed and used to satisfactorily solve the MDVRP with efficient approximation of the optimal solution. Pisinger and Ropke [39] proposed a unified heuristic, which was able to solve five different variants of the vehicle routing problem by using the Adaptive Large Neighborhood Search (ALNS). The authors employed a number of insertion and removal heuristics, to intensify and diversify the ALNS search process. In their study, five different variants of the VRP were considered, namely VRPTW, CVRP, MDVRP, the site dependent vehicle routing problem (SDVRP) and the open vehicle routing problem (OVRP). Experimental results from the study [39] show that the proposed algorithm improved 183 best known solutions out of the 486 benchmark tests.

Vidal *et al*. [30] developed a hybrid genetic algorithm framework for solving the MDVRP and periodic VRP. The proposed hybrid algorithm combines the advantages of the exploration breadth of population-based evolutionary search process, the aggressive improvement capabilities of the neighborhood-based methods, and the advanced population diversity management scheme to improve its solution quality and robustness. The proposed method was tested on standard VRP benchmark instances. Experimental results conducted demonstrate the superiority of the hybrid algorithm in terms of computational efficiency and solution accuracy. Since the new method was able to efficiently identify the best known solutions, including the optimal solutions and new solutions for all the tested benchmark instances, it was concluded that the algorithm was better than the compared techniques.

Juan *et al*. [10] combined biased randomization with iterated local search to develop a hybrid method for solving the MDVRP with a limited number of identical vehicles per depot. Biased randomization is the use of non-symmetric probability distributions to generate randomness. In [10], two biased-randomized processes were employed at different stages of the iterated local search framework in order to assign customers to depots and also to improve routing solutions. One advantage of their method is that the hybrid algorithm utilizes only one parameter and as such, does not require any parameter tuning. The proposed hybrid algorithm by Juan *et al*. [10] was tested on a standard benchmark with great success, which makes it an interesting approach for solving MDVRP.

In a related work by Teymourian *et al*. [29], a framework consisting of enhanced intelligent water drops and cuckoo search algorithms was proposed for solving the capacitated vehicle routing problem. The framework employs four state-of-the-art algorithms to solve the CVRP. These algorithms include an improved IWD algorithm, advanced cuckoo search algorithm, two local search hybrid algorithms, and a post-optimization hybrid algorithm. The proposed hybrid approach takes merit of the capabilities of the two metaheuristics (that is, the improved IWD and advance cuckoo search algorithms) to explore and exploit the solution search spaces. The performance of the proposed algorithms was evaluated on two well-known benchmark instances from literature and their results were compared with some other state-of-the-art algorithms from literature. The experimental results show that proposed methods can effectively cope with large-scale problems, where in most cases the local search hybrid algorithm yields the best gained solutions in the literature.

## Metaheuristic solution to MDVRP

The two metaheuristic algorithms employed to develop the proposed hybrid algorithm, are discussed in this section. The IWD metaheuristic algorithm is discussed firstly, followed by the description of the approximate SA algorithm.

### Intelligent water drops algorithm

The IWD algorithm is a population-based evolutionary metaheuristic algorithm influenced by the movement of natural water drops finding its way to the river, lakes, or seas. The idea of the IWD algorithm was first proposed by Shah-Hosseini [32] in 2009. Since then, the algorithm has been applied to solve several optimization problems, such as the n-queen puzzle multidimensional knapsack problem [33], multilevel thresholding of gray-level images [33], multi-objective job shop scheduling in scheduling system [40], optimum reservoir operation in water resources systems [41], robot path planning in robotics [42], economic load dispatch problem in power systems [43], feature selection with rough set [44], search and selection optimization processes [45], and examination time-tabling scheduling problem [46]. (Reference could be made to [47] for a comprehensive summary of the various problems that have been successfully solved using the IWD algorithm).

**Fig 2:**
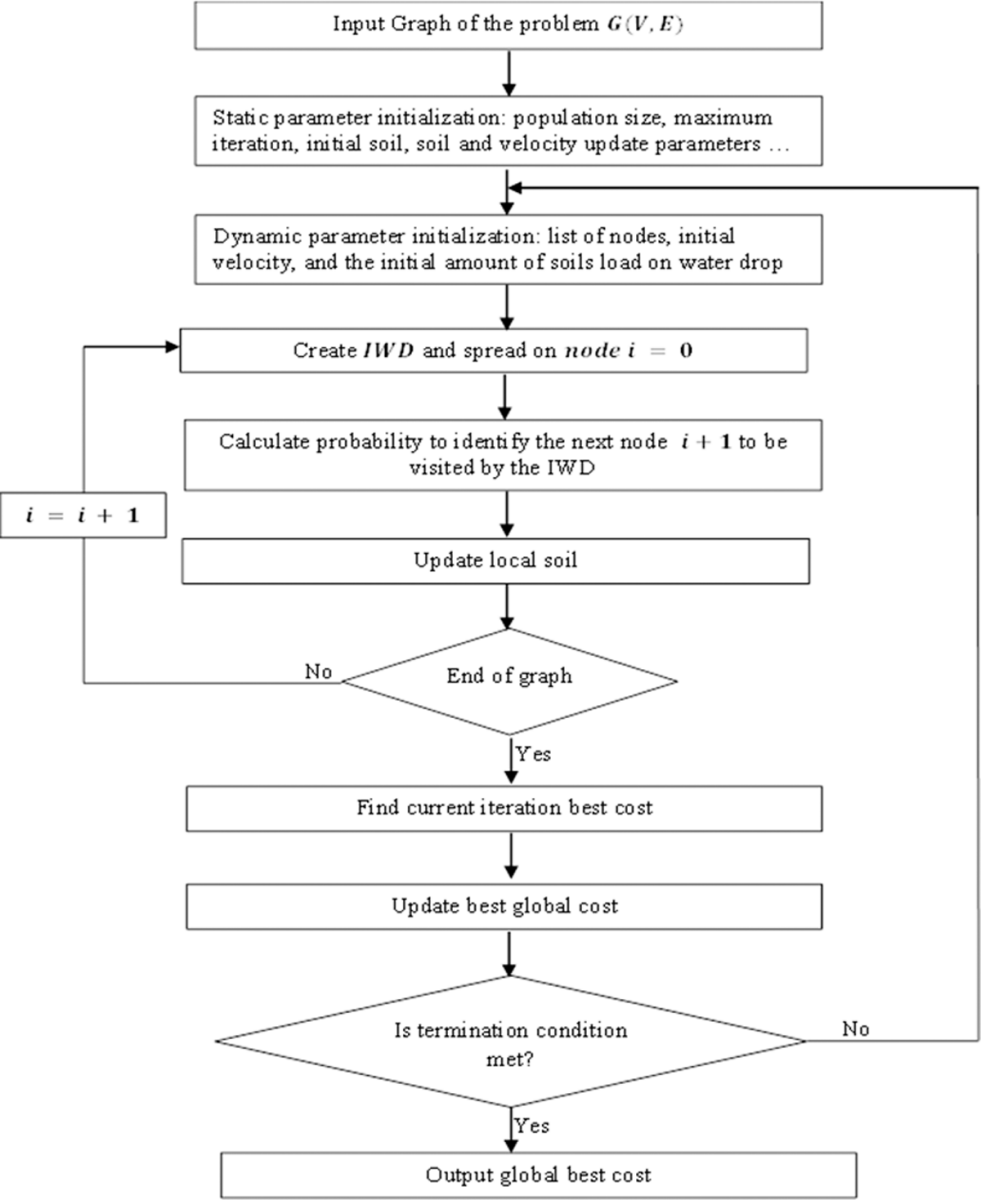
Flow diagram of the proposed IWD.

The IWD algorithm problem solving approach is modeled in the form of a graph, *G* = (*V*, *E*), where *V* and *E* denote sets of nodes and edges. The structure of the graph depends on the problem representation. The orientation of the problem for which the IWD algorithm is supposed to optimize and find a solution, is usually viewed based on the assumption that there exists a node source from which the water drop moves through a selected path to the next unvisited node. The paths through which the water drops traverses have some loads of soils. Therefore, the choice of selecting a specific path by the IWD is dependent on the amount of soil present on the unvisited path and the path with less soil is usually selected. However, during this process the velocity of the water drops may change, depending on the quantity of soil it is able to offset or accumulate along the selected path of movement. The whole process is repeated iteratively and the best path is updated periodically until the best solution or global solution is found and updated subsequently, after which the algorithm is terminated. The main algorithm procedure and the flowchart are presented in algorithm listing 1 and Fig 2.

**Algorithm listing 1**: Basic IWD algorithm steps

**Input**: Graph *G* = (*V*, *E*) where *V* and *E* are sets of nodes and edges

**Output**: global best solution

Parameter initialization:

Set static parameters: population size, maximum iteration, initial soil, soil update parameters and velocity update parameters

1. ***Do***
2. Initialize the dynamic parameters: IWDs, list of visited nodes, initial velocity, and the initial amount of soils load on water drop
3. Construct solutions by IWDs
4. Search for the current best solution
5. Update the soils path that forms the current best solution
6. Update the best solution
7. ***While*** (termination condition is not met)
8. Return the best solution

There are two types of parameter settings defined for the IWD algorithm, namely static and dynamic parameter settings. Some examples of the static parameters include termination criteria, which determine when the algorithm should be terminated, the initial soil paths and velocity update parameters, which are constant throughout the iterative execution of the algorithm. On the other hand, examples of the dynamic parameters include a list of visited nodes, and the initial amount of soil load on water drop. This type of parameters changes its values with every increment in iteration steps.

### Intelligent water drops algorithm for solving MDVRP

To successfully apply the IWD algorithm to solve the MDVRP, the structure or environment of the MDVRP which is denoted by *G* = (*N*, *E*) must be defined. Applying the steps presented in this section help minimize the total dispatching cost of each assigned vehicle that is supposed to service a customer. To start with, a graph with *n* nodes and *n*(*n*−1)/2 directed edges are constructed. This is used by the IWDs as input to construct solutions for the optimization process. In this case a node denotes a customer, while an edge denotes a route to a customer. Every IWD begins its journey by starting from the first or initial node *i* and terminates it by visiting the end node *i* + 1 on the graph. Therefore, some of the important factors and parameters employed by the IWD algorithm to solve the optimization process of the MDVRP are discussed.

### Parameter initialization

The first step in the solution construction phase is the initialization of all the static and dynamic parameters such as the number of water drops, which in the current case is equal to the number of vehicles, each edge’s soil and each IWD’s velocity. The static parameters remain constant throughout the program run, and examples of the static parameters include number of IWDs, the initial value of soil, maximum number of iterations, velocity and soil updating parameters. The dynamic parameters are usually initialized at the start of the IWD search process and are subsequently updated throughout the search process. Examples of the dynamic parameters include a list of nodes visited by IWD (which in this case is vehicle k), initial velocity of IWD, and the initial soil load on IWD.

#### Route building

Route design by the IWD algorithm is such that at each iteration phase of the algorithm, the individual IWDs build a solution for the MDVRP by moving from customer *i* to the next customer *i* + 1 according to a defined selection rule, which is explained below. This process is usually evaluated based on the amount of soil present at each customer’s node and the route distance to that node. Each IWD is able to keep track of the list of nodes visited. It is equally important to mention here that route construction for all the available customers is done in parallel by the IWDs. For each of the IWD iterations, only one customer is selected by using the probability distribution function given in Eq 1 below. In the proposed IWD implementation, the route building process executed by the IWDs is based on a parallelism processes adopted by the algorithm to construct its solution.

#### Solution representation

**Fig 3:**
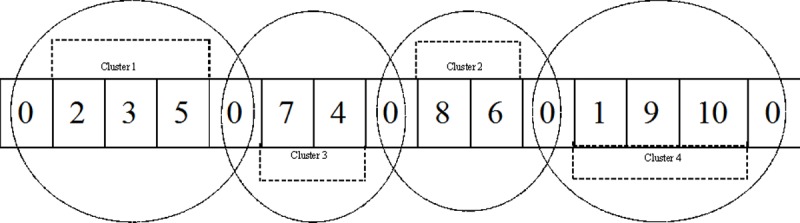
Solution representation scheme with indication of clustering.

Building a solution for the MDVRP as stated earlier, consists of two stages, namely clustering of customers and routing for each cluster. To retain the simplicity of the IWD algorithm, a straightforward solution representation scheme is employed, which has also been used in existing research [29, 48]. For the solution representation mechanisms, a single string is used to represent the clustering and routing stages. The clustering process is formulated by computing the distance matrix given in Eq 8, which is based on the steps described in the multi-depot vehicle routing problem section above. In Fig 3, a solution representation of a typical MDVRP solution scheme is illustrated, where three vehicles (*k* = 3) serve ten customers (*n* = 10), and the depot in this case is represented by 0. In this solution representation, customers 2, 3 and 5 are assigned to the same vehicle, which is vehicle 1, and the routing sequence is the same as the customer order, which is in the sequence of customer 2 first, customer 3 second, followed by customer 5. The second vehicle (vehicle 2) visits customers 7 and 4, for which the routing sequence is customer 7 first, then customer 4. The same goes for vehicle 3 that visits customers 1, 9, and 10, with the routing sequence the same as the customer order. The resulting clusters as shown in Fig 3 consist of some list of customers and the advantage of the clustering process is to generate optimum routes for the allocation of different vehicles to customers. In addition, since clustering in this case can be treated as a local search process, the SA algorithm can be employed as a local search metaheuristic to determine the best cost required for each vehicle to travel to a customer in the same cluster. Therefore, a fitness function, which is defined as the total distance covered by each vehicle per cluster, can be used to evaluate the quality of IWD solution. The implementation of SA is detailed in subsequent section below.

**Fig 4:**
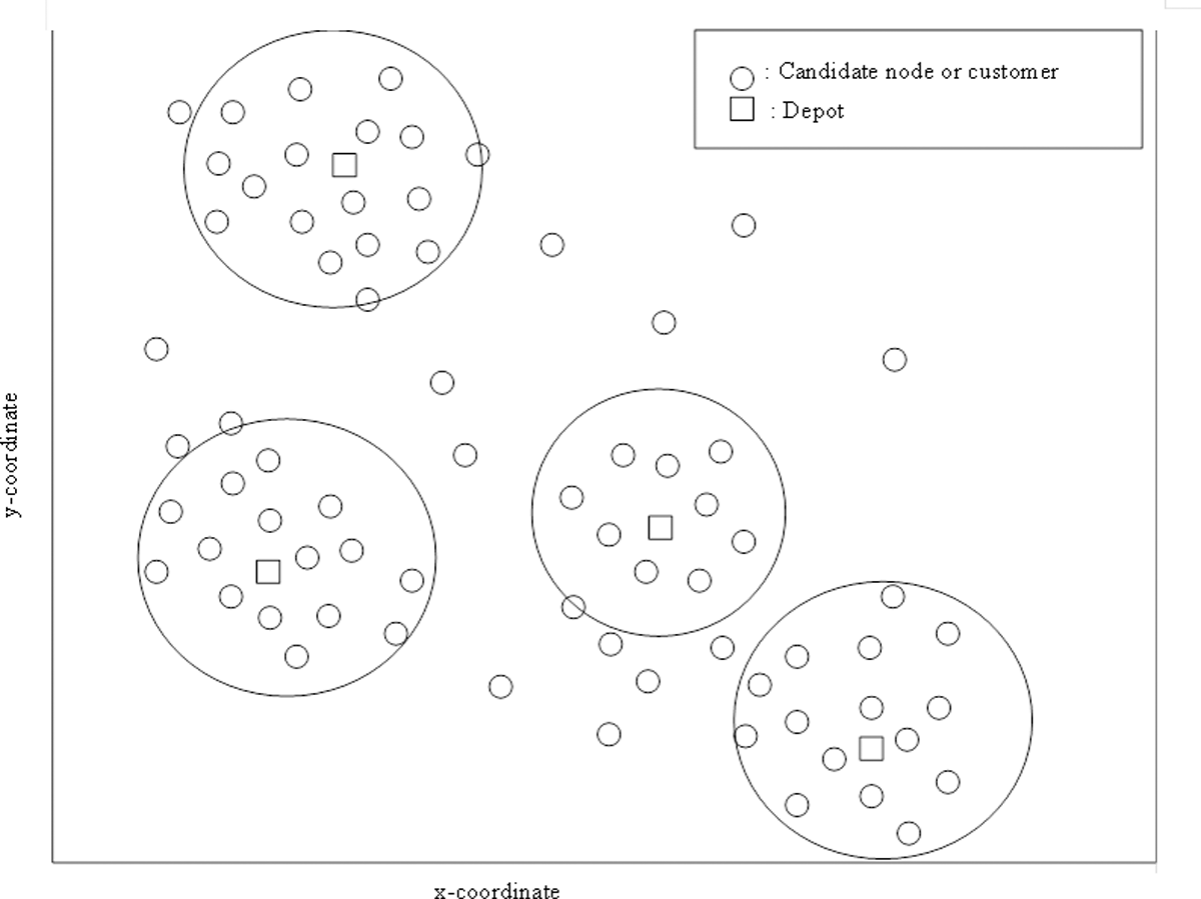
Example illustration of node cluster construction by IWD.

The main goal of the solution construction phase is to generate a population of IWD feasible solutions for MDVRP by implementing the IWD hybrid method described in algorithm listing 3 below. At the initial stage of the algorithm implementation, each IWD starts from a depot and selects the next customer to visit within its mapped cluster that consists of a list of candidate customers. The vehicle constraints are updated after the first visit and if these constraints are not violated, the second customer is selected by the IWD. More so, considering the VRP solution representation presented in Fig 3, each individual IWD generate a cluster of feasible solution for the MDVRP by simulating a vehicle and constructing its routes by iteratively selecting customers based on the vehicle’s predefined constraints of not exceeding its capacity and visiting each customer once, until all the customers in the feasible solution search spaces (as shown in Fig 4) are serviced. Each IWD follows a similar approach to construct a feasible route for all vehicles. The construction phase is completed by the transition of all IWDs through the feasible solutions, namely that once the vehicle constraints are satisfied and all the customers having been visited, the process is terminated with the IWDs returning back to depot. The solution construction phase is modeled based on an undirected graph with edges and nodes. Therefore, each IWD transverses along the edges from its nodes according to a certain defined probability function explained in the next section.

#### Selection rule

The selection rule is employed by IWD to select an optimum path *i* + 1 to a customer or node according to the probability $P_{i}^{IWD}\left(i+1\right)$. In the proposed optimization method, each IWD utilizes a probability distribution function assigned along each edge starting from the current node *i* through to all other nodes, *i* + 1, which do not violate the assigned constraints of the problem under consideration. The selection mechanism for choosing an edge connected to the next node by the IWD, is expressed as follows: Let an IWD be at node *i*, then the probability distribution denoted by $P_{i}^{IWD}$, which is required by IWD to select an edge to move from node *i* to node *i* + 1, is calculated using a fitness function as expressed in Eq (1) as follows.

$$P_{i}^{IWD}\left(i+1\right)=\frac{f\left(soil\left(i,i+1\right)\right)}{\sum_{n\notin C}f\left(soil\left(i,n\right)\right)}$$ (1)

$$f\left(soil(i,i+1\right))=\frac{SM\left(i,i+1\right)}{\varepsilon +g\left(soil\left(i,i+1\right)\right)}$$ (2)

SM(i,i+1)=d(h, i)+d(h,i+1)−d(i,i+1) (3)

$$d\left(i,h\right)=\sqrt{{\left(x_{i}-x_{h}\right)}^{2}+{\left(y_{i}-y_{h}\right)}^{2}}$$ (4)

where the function *SM*(.) is the saving matrix proposed by [49], which is used to compute the distance traveled by the IWDs along the edges to visit the nodes or as in the current case, the distance traveled by the vehicles for serving the customers. Here, the saving matrix is constructed for every two customers *i* and *i* + 1, on the same link path to the given depot *h*. The parameter ε is a very small positive number assigned to prevent singularity or possible division by zero. The function *g*(*soil*(*i*,*i*+1)) serves as shift function that moves the soil through an edge joining any two nodes, *i*+1 towards a positive value. The function is given as follows:

$$g\left(soil\left(i,i+1\right)\right)=\{\begin{array}{c} soil\left(i,i+1\right)if\underset{n\notin C}{\min}\left(soil\left(i,n\right)\right)\geq 0\\ soil\left(i,i+1\right)-\underset{n\notin C}{\min}\left(soil\left(i,n\right)\right)\;otherwise \end{array}$$ (5)

where *C* denotes the set of nodes that IWD is not allowed to visit.

Therefore, the edge selection procedure between two nodes *i* + 1 and *k* from node can be summarized based on the following conditions: If $P_{i}^{IWD}\left(i+1\right)<P_{i}^{IWD}\left(k\right)$, then select edge k to visit the connected node If $P_{i}^{IWD}\left(i+1\right)>P_{i}^{IWD}\left(k\right)$, then select edge *i* + 1 to visit the connected node If $P_{i}^{IWD}\left(i+1\right)=P_{i}^{IWD}\left(k\right)$, then select edge arbitrarily to visit any of the connected node

#### Set of visited nodes

An updated list of visited paths *C* created earlier on, is maintained by IWD as a means of keeping track of all the visited nodes. Iteratively, each node is first evaluated on the basis of whether it has been visited or not, before a decision is taken. If it is confirmed that the node has been visited previously, the node is deleted. The check is performed in order to prevent the IWD from traversing the same path twice. Relating this technique to the MDVRP for example, having customer *i* on the route of vehicle *k* from depot *h*, and after *i* has been visited by *k*, it is removed completely from that route and any subsequently visit by *k* to *i* is declared taboo for a specific number of iterations. This condition holds for as long as *k* is only allowed to serve *i* just once.

#### Local soil and velocity update

As IWD move from node *i* to node *i* + 1, its velocity is updated as follows:

$$vel^{IWD}\left(t+1\right)=vel^{IWD}\left(t\right)+\frac{a_{v}}{b_{v}+c_{v}.soil\left(i,i+1\right)}$$ (6)

where *a*_*v*_, *b*_*v*_ and *c*_*v*_ are the IWD velocity updating parameters and *vel*^*IWD*^ (*t*) is the previous velocity of the IWD.

The time taken by IWD to move from node *i* to node *i* + 1 is computed as follows:

$$Time\left(i,i+1;vel^{IWD}\right)=\frac{d\left(i,i+1\right)}{\text{max}\left(\varepsilon_{v},vel^{IWD}\right)}$$ (7)

$$d\left(i,i+1\right)=\sqrt{{\left(x_{i}-x_{i+1}\right)}^{2}+{\left(y_{i}-y_{i+1}\right)}^{2}}$$ (8)

The *max*(.,.) returns the maximum value between its arguments’. This value is then used to threshold the negative velocities to a very small positive number ε_v_. The function *d*(.) represents the distance taken by IWD to move from node *i* to node *i* + 1.

The amount of soil on the link will be reduced, since IWDs carries some soil as it traverses from node *i* to node *i* + 1. Therefore, the soil carried by the IWD as it moves along the link from node *i* to node *i* + 1 is updated as follows:

Soil(i,i+1)=(1−ρ).soil(i,i+1)−ρ.Δsoil(i,i+1) (9)

$$soil^{IWD}=soil^{IWD}+\Delta Soil\left(i,i+1\right)$$ (10)

$$\Delta Soil\left(i,i+1\right)=\frac{a_{s}}{b_{s}+c_{s}.Time\left(i,i+1;vel^{IWD}\right)}$$ (11)

where *a*_s_, *b*_s_, and *c*_s_ are the IWD soil updating parameters, ρ is a small positive number (0<ρ<1).

#### Fitness function

The main reason for determining the fitness function is to increase the chances of finding a global-best solution and to also improve the convergence speed of the IWD algorithm. The fitness function determines the ranking of the individual solution obtained by calculating the total length of the constructed route traversed by each IWD in the iterations. The solution with the minimum route length among all the IWD constructed routes is then taken as the best solution. Since this is a minimization case, the route length denoted by *T*^*IWD*^ can be expressed as follows:

$$T^{IWD}=\overset{n-1}{\underset{i=1}{\sum }}d\left(T_{i}^{IWD},T_{i+1}^{IWD}\right)+d\left(T_{n}^{IWD},T_{1}^{IWD}\right)$$ (12)

Therefore, the fitness function can be defined as follows:

$$f=min\left(\underset{\forall T^{IWD}}{\sum }\left(T^{IWD}\right)\right)$$ (13)

where *n* is the total number of nodes or customers and the function *d*(.) is the Euclidean distance between customer *i*s and customer *i* + 1.

#### Global update

To prevent IWD from plunging into local minima, the amount of soil on each of the current iteration’s best solution with the minimum route length *T*_M_, was subsequently updated as follows:

$$Soil\left(i,i+1\right)=\left(1-\rho \right).soil\left(i,i+1\right)+\rho .\frac{2.soil^{IWD}}{n\left(n-1\right)}\forall \left(i,i+1\right)\in T_{M}$$ (14)

However, if at the end of each iteration process, *T*_M_ is found to be shorter than the current best solution, which is denoted by *T*_B_, the best route is updated as follows:

$$T_{B}=\{\begin{array}{c} T_{M}\;iff(T_{B})\geq f\left(T_{M}\right)\\ T_{B}\;otherwise \end{array}$$ (15)

#### Termination condition

The program is terminated once there is no further improvement on the global soil updating, that is after a number of successive iterations have been performed. This also corresponds with the value of the constant parameter referred to as the maximum number of iteration, which is set to 100 in the case of the proposed method.

### Simulated annealing

Since its introduction as a solution method into the field of optimization techniques, SA algorithm has been used to solve numerous optimization problems, either on the basis of it being used as a classical algorithm or as part of a hybrid implementation when combined with other metaheuristics. As a local search metaheuristic, the application of SA has produced fairly good results in comparisons with other heuristic based algorithms [50, 51]. The SA algorithm is intensely studied in the literature and in some cases it is specifically applied to solve the VRP and its variants problems [20].

**Fig 5:**
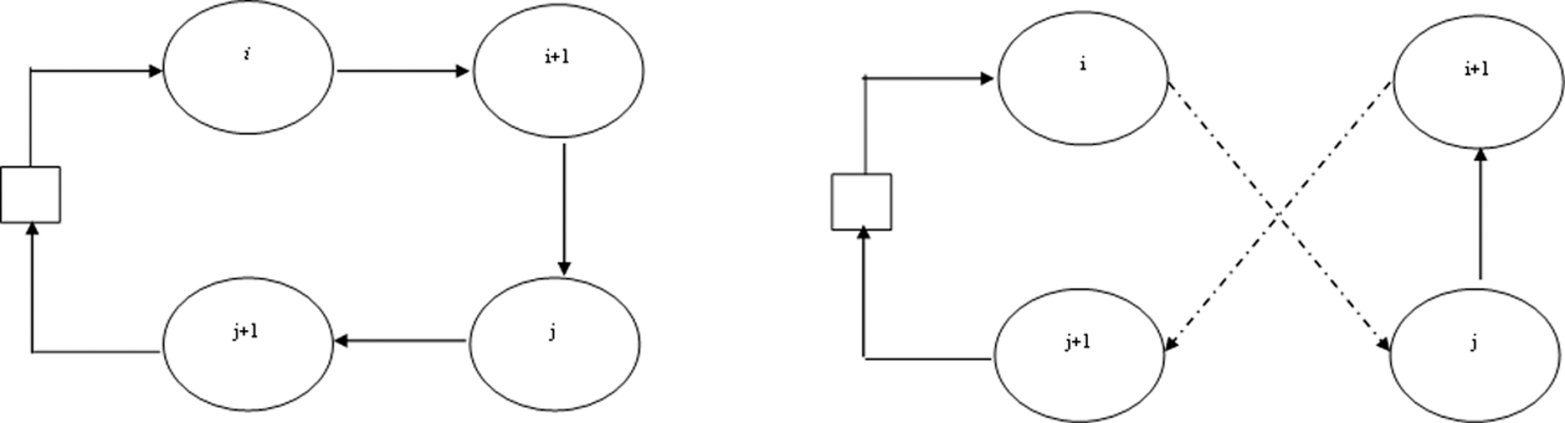
Illustration of 2-opt operator as applied to SA.

The reason for the introduction of SA into IWD was to enhance the existing solution quality of the results produced by the IWD. Another reason for the combination of these two algorithms was to develop a robust local search technique that prevents the IWD from getting stuck into local minima as earlier discussed in the clustering process presented above. SA can be viewed as a search process that can always attempt to move from one current solution to another solution in its neighborhood solutions. Therefore, it has the potential of providing better objective values for the IWD to solve the MDVRP efficiently. Unlike the hill climbing method, SA is able to escape from getting trapped into local minima by allowing worse moves (lesser quality) or uphill steps to be taken at random some of the time. SA choice of selecting best solution is based on its movement procedure, which is such that if the anticipated move is better than its current position, then SA will always take it. If the move is worse, it will be accepted based on some probability selection criteria. For the MDVRP, the SA procedure begins by considering the solution construction of each IWD, given as $T_{i}^{IWD}|i=1,2,\ldots ,n$, for a set of given customers with an updated solutions $T_{i+1}^{IWD}$ created by randomly switching the orders of two customers. The switching order is implemented based on the concept of 2-opt exchanges of paths between different customers’ routes [52, 53]. In the 2-opt operator, all possible pairwise exchanges of customers inside an individual vehicle route are evaluated to see if a shorter route distance can be obtained by exchanging the order in which the customers are visited by the vehicle. To briefly illustrate this concept as shown in Fig 5, the following nodes are considered {*i*, *i*+1} and {*j*, *j*+1} and the process is represented as follows: {*i*, *i*+1}{*j*, *j*+1}→{*i*,*j*}{*i*+1,*j*+1}. In Fig 5, the rectangle represents a depot, while the circles denote customers or nodes.

The cost function or fitness function, which represents the quality of solution $T_{i}^{IWD}$ (that is the total distance or route length travelled by the vehicles in a cluster) is denoted by $f(T_{i}^{IWD})$. The relative change in cost Δ*f* between $T_{i}^{IWD}$ and $T_{i+1}^{IWD}$ is expressed as $\Delta f=f\left(T_{i+1}^{IWD}\right)-f\left(T_{i}^{IWD}\right)$. Since the fitness functions of the solution are computed iteratively, an approximate evaluation of the function that at the same time provides a good indication of the solution quality can be employed. It is equally important to note that a simplified evaluation of the fitness function is necessary to reduce the computation time of the SA.

Beginning with the initial solution, only the solution which has a smaller fitness value than the previous solution is accepted by the algorithm. In other words, a solution is only accepted with the fitness value of $f(T_{i+1}^{IWD})<f\left(T_{i}^{IWD}\right)$. However, accepting or rejecting a new solution with higher fitness values for $T_{i+1}^{IWD}$ is based on the probability of acceptance criteria given as follows:

$$p\left(f,t_{\tau }\right)=exp\left(-\frac{f\left(T_{i+1}^{IWD}\right)-f\left(T_{i}^{IWD}\right)}{kt_{\tau }}\right)$$ (16)

In the case of large problem sizes, instead of using Eq 16, Eq 17 can be employed to improve the performance of the SA:

$$p\left(f,t_{\tau }\right)=exp\left(-\frac{f\left(T_{i+1}^{IWD}\right)-f\left(T_{i}^{IWD}\right)}{t_{\tau }}\right)$$ (17)

Similarly, Eq 17 can be approximated to Eq 18. This approximation method is applied in this case to reduce the computational effort or time of the SA procedure.

$$p\left(f,t_{\tau }\right)=1-\left(\frac{f\left(T_{i+1}^{IWD}\right)-f\left(T_{i}^{IWD}\right)}{t_{\tau }}\right)$$ (18)

where *t*_τ_ is the parameter temperature at the τ^*th*^ instance of accepting a new solution. The probability of accepting a new solution is a function of both the temperature of the system and the difference in the fitness value. It has been noted that the probability of accepting a worse solution decreases as the temperature deteriorates. This means that as the temperature reduces to zero, only better solutions will be accepted. In this paper, the following cooling schedule is adopted (Eq 19):

$$t_{\tau +1}=\alpha t_{\tau }$$ (19)

where, α denotes the rate at which the temperature is lowered each time a new solution $T_{i+1}^{IWD}$ is discovered. The SA procedure is presented in the algorithm listing 2. Therefore, in this paper, two versions of the hybrid methods are proposed namely, the hybrid method that combines IWD algorithm and SA algorithm using Eq 17, which is denoted here as IWD-SA, and the second proposed hybrid method that combines IWD algorithm with approximated SA algorithm using Eq 18, which is denoted here as IWD-ASA.

**Algorithm 2:** Pseudocode for local search implementation by SA

**Input**: Initial temperature *t*_0_, final temperature *t*_#x03C4;_, cooling rate α, maximum iteration *maxiter*

**Output**: Best cost per cluster

1. Identify a solution $T_{i}^{IWD}$ from clusters consisting of random solutions $T_{i}^{IWD}|i\;=\;1,2,\ldots ,n$
2. Do
3. *For counter* = 1 *to maxiter*
4. Create a new solution $T_{i+1}^{IWD}$ from $T_{i}^{IWD}$
5. ***If*** $f\left(T_{i+1}^{IWD}\right)\leq f\left(\;T_{i}^{IWD}\right)$ ***then***
6. $T_{i}^{IWD}\leftarrow T_{i+1}^{IWD}$
7. ***Else***
8. ***If*** *p* > r(0,1) ***then*** (using either Eq 17 in terms of IWD-SA or Eq 18 in terms of IWD-ASA) where *r* is a randomly generated number
9. $T_{i}^{IWD}\leftarrow T_{i+1}^{IWD}$
10. ***End if***
11. Reduce the temperature using Eq 19 and increment τ = τ +1s
12. Update the best solution
13. ***End for***
14. ***While*** (termination condition is not met)

### The proposed hybrid method

**Fig 6:**
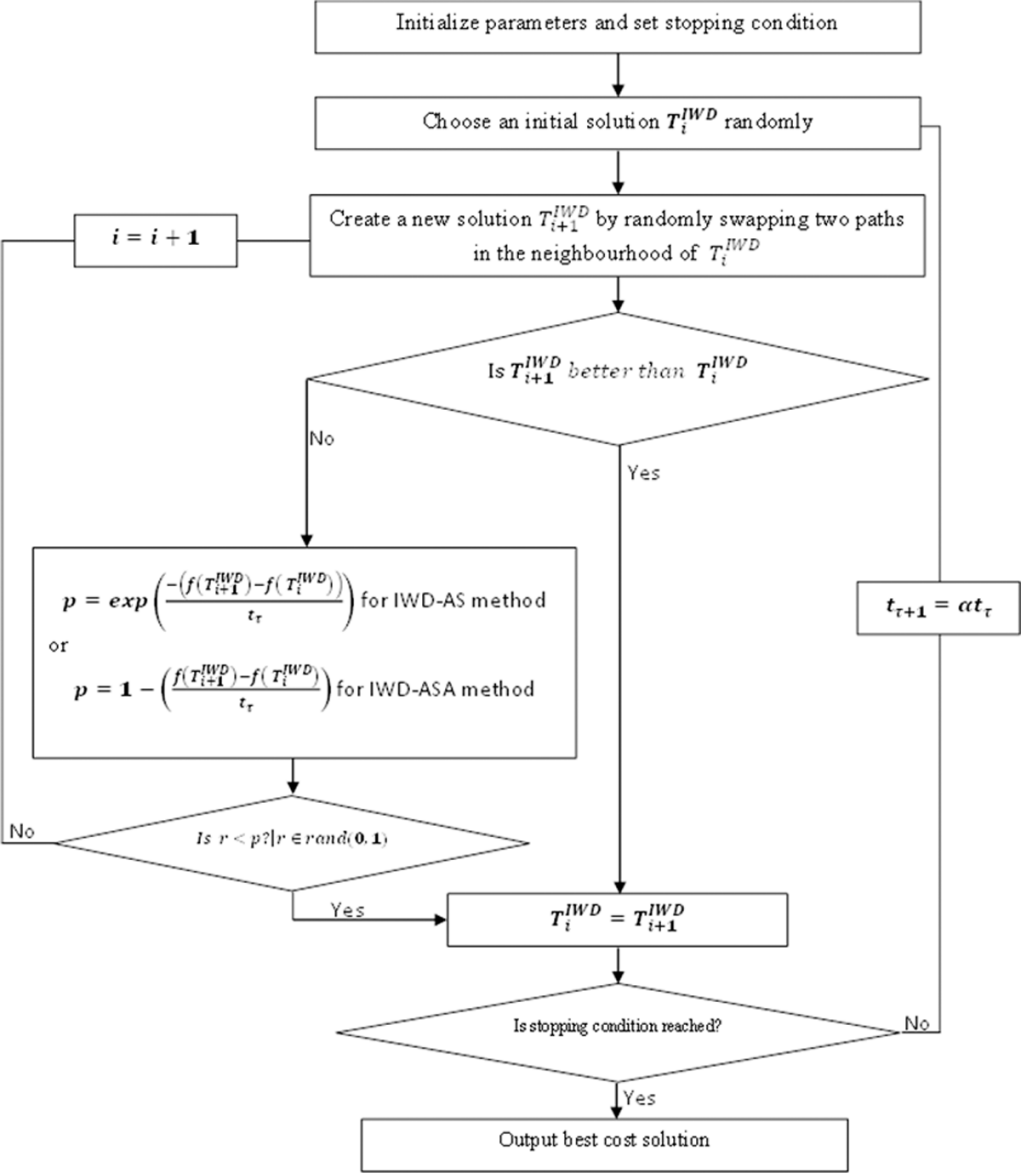
Flow diagram of the proposed SA based IWD.

The hybrid IWD-SA algorithm introduces the SA probability of acceptance criteria in determining between the current best cost and the new solution cost, which of them to choose as the solution to be updated. The algorithm achieves this technique by computing and comparing iteratively the quality of solution obtained by both the old and new IWD’s drops, which revolve around *T*_*M*_ and *T*_*B*_. Therefore, the acceptance rule is evaluated based on two conditions namely, the fitness function or quality of solution, and the environmental temperature. The acceptance criteria enable the IWD process to easily escape from being trapped into local minima, thereby increasing the rate of the algorithm exploration and convergence. The step by step description of how the combined IWD algorithm and SA method work together to achieve the aforementioned process, is presented in Algorithm listing 3. The illustration of these steps is also illustrated using the flowchart shown in Fig 6.

**Algorithm listing 3**: IWD-SA algorithm steps

**Input**: Graph *G* = (*V*,*E*)where *V* and *E* are set of nodes and edges

**Output**: global best solution

Parameter initialization:

Set static parameters: population size, maximum iteration, initial soil, soil update parameters and velocity update parameters

1. ***Do***
2. Initialize IWDs, list of visited nodes, initial velocity of water drop, and the initial amount of soils load on water drop
3. ***For*** *k = 1 to N*_*c*_ where *N*_*c*_ is the number of nodes on the graph
4. Search for the current best solution by spreading the IWDs randomly on the problem graph
5. Update all the dynamic parameters to include: list of visited nodes, initial velocity of the water drop and the initial amount of soil loaded onto water drop
6. ***End for***
7. Create a new solution $T_{i+1}^{IWD}$ from $T_{i}^{IWD}$
8. Calculate the fitness functions $f\left(T_{i}^{IWD}\right)$ and $f(T_{i+1}^{IWD})$
9. ***If*** $f\left(T_{i+1}^{IWD}\right)\leq f\left(T_{i}^{IWD}\right)$ ***then***
10. $T_{i}^{IWD}\leftarrow T_{i+1}^{IWD}$
11. ***Else***
12. Calculate *p*(*f*,*t*_τ_) with Eq 17 for IWD-AS method and Eq 18 for IWD-ASA method
13. ***If*** *p*>r(0,1) ***then***
14. $T_{i}^{IWD}\leftarrow T_{i+1}^{IWD}$
15. ***End if***
16. Update the soil value of all paths visited by the *IWD* ∈ *T*_M_ Update the global best cost *T*_B_ by comparing the fitness values of *T*_M_ and *T*_B_
17. ***If*** (*fT*_B_ ≥ *fT*_M_) ***then***
18. *T*_B_=*fT*_M_
19. ***Else***
20. *T*_B_=*T*_B_
21. ***End if***
22. Update temperature *t*_k+1_ = α*t*_k_
23. ***While*** (termination condition is not met)
24. Return the best cost *T*_B_

## Computational experiment

This section presents the various experimental assessments of the IWD algorithms demonstrated in three phases. The first phase is the experiment phase that combines the computational effort of both the IWD algorithm and SA to find the minimum best cost through the optimization of the MDVRP, which is achieved by using Eq 17. The second phase of the experiment demonstrates the results obtained by combining IWD algorithm and the approximate version of the SA algorithm using Eq 18. The last phase of the experiment demonstrates the application and effectiveness of IWD algorithm to solve the MDVRP. The objective of this evaluation is to report the variations in the results emanating from the improvements of the IWD algorithm based on the three empirical scenarios. The results in each method are compared with the best known solution from Cordeau’s instances taken from Cordeau *et al*., [37]. The three experiments were set up and executed under the same processing conditions. The three case scenarios of the IWD and SA algorithms were executed in Windows 7 OS, Intel Celeron CPU@1.8GHz with 3.88GB of RAM. MATLAB R2014b was used as the programming language. The MDVRP instances results described in Cordeau *et al*. [37], Pisinger and Ropke [39], Vidal *et al*. [30] and Juan *et al*. [10], were benchmarked to evaluate the performance of the three methods proposed in this paper. Each instance of the problem was run 10 times for a maximum number of 100 iterations.

The parameters used for the implementation of the proposed methods are as presented in Table 1. In the course of the experiment, it was observed that the choice of parameter selection sometimes influences the quality of solution obtained, the higher the values of the parameters being selected, the more computation time it consumes in some cases. This is true in the case of the simulated annealing with an exponential calculation of the probability of acceptance criteria that is computationally expensive. In the analysis of the three methods described in this paper, both the static and dynamic parameters are configured as shown in the parameter Table 1 for the IWD and Table 2 for the SA algorithm. The static parameters are set using similar theoretical values from the work presented in [32].

**Table 1:** The parameter values for IWD procedures.

Parameter	Values
Initial soil	1000
Initial velocity	100
Maximum number of iterations	100
IWD soil updating parameters *a*_*s*_	1000
IWD soil updating parameters *b*_*s*_	0.01
IWD soil updating parameters *c*_*s*_	1
IWD velocity updating parameters *a*_*v*_	1000
IWD velocity updating parameters *b*_*v*_	0.01
IWD velocity updating parameters *c*_*v*_	1

**Table 2:** The parameter values for SA procedures.

Parameter	Values
Initial temperature *t*_*0*_	15
Final temperature *t*_*τ*_	0.001
cooling coefficient *α*	0.99

The first experiment conducted aimed to compare between IWD algorithm and the two versions of the SA, that is, SA with the exponential calculation of the acceptance probability function (IWD-AS) and with an approximate version of the exponential probability of acceptance criteria (IWD-ASA). The results of these tests are summarized in Table 3. Moving column wise in Table 3, the best known solution (BKS) for the Cordeau MDVRP instances is shown, while the average results for the 10 runs is denoted by ‘AVE’, the best solution found is denoted by ‘Best’, the standard deviation is denoted by ‘Std. Dev’, and the average CPU time in seconds is denoted by ‘Time’ are equally shown.

**Table 3:** Computational results obtained for 33 Cordeau MDVRP benchmark instances for improved IWD, IWA-SA, and IWD-ASA.

INSTANCE					IWD					IWD-SA						IWD-ASA					
**NO**	**Inst.**	n	h	BKS	AVE	Best	Std. Dev	Time	Gap (%)	AVE	Best	Std. Dev	Time	Gap (%)	t-test	AVE	Best	Std. Dev	Time	Gap (%)	t-test
1	P01	50	4	576.87	576.87	**576.87**	0.00	13.66	0.00	576.87	**576.87**	0.01	14.20	0.00	0.00	576.87	**576.87**	0.01	14.299	0.00	0.00
2	P02	50	4	473.53	491.17	**473.53**	0.48	13.55	0.00	479.03	**473.53**	0.07	14.02	0.00	79.14	476.53	473.53	0.05	13.92	0.00	95.93
3	P03	75	5	641.19	648.19	643.58	1.58	41.93	0.37	641.19	641.19	0.03	43.62	0.00	14.01	641.19	641.19	0.02	43.192	0.00	14.00
4	P04	100	2	1001.59	1010.23	1006.19	1.05	96.31	0.46	1002.23	1001.59	0.68	99.43	0.00	20.22	1002.06	1001.59	0.52	96.693	0.00	22.05
5	P05	100	2	750.03	758.13	754.84	1.01	95.13	0.64	752.13	750.03	0.52	99.87	0.00	16.70	752.13	750.03	0.71	95.349	0.00	15.37
6	P06	100	3	876.50	888.49	879.71	2.45	95.22	0.37	881.49	876.50	1.05	104.21	0.00	8.30	881.49	876.50	1.23	96.10	0.00	8.07
7	P07	100	4	885.80	901.83	885.82	4.80	95.97	0.00	889.12	885.80	2.04	105.61	0.00	7.71	889.12	885.80	2.17	96.369	0.00	7.63
8	P08	249	2	4437.68	4572.56	4492.39	24.56	1369.40	1.23	4430.48	**4430.48**	0.66	1414.80	−0.16	18.29	4430.48	**4430.48**	0.61	1389.01	−0.16	18.29
9	P09	249	3	3900.22	3942.81	3910.62	11.24	1371.21	0.27	3912.81	3900.10	4.69	1420.30	0.00	7.79	3912.81	3900.10	4.51	1391.56	0.00	7.83
10	P10	249	4	3663.02	3689.54	3663.29	7.44	1370.10	0.01	3662.54	**3659.84**	0.88	1422.04	−0.09	11.40	3662.54	**3659.84**	0.74	1390.78	−0.09	11.42
11	P11	249	5	3554.18	3695.27	3565.17	4.72	1368.10	0.31	3601.72	**3552.34**	7.89	1489.67	−0.05	32.18	3616.32	**3552.34**	5.30	1392.30	−0.05	35.18
12	P12	80	2	1318.95	1376.03	1324.34	16.63	50.97	0.41	1321.41	1318.95	1.51	54.91	0.00	11.93	1321.41	1318.95	1.56	51.502	0.00	10.34
13	P13	80	2	1318.95	1353.95	1324.05	8.81	50.15	0.39	1318.95	1318.95	0.52	55.05	0.00	12.54	1318.99	1318.95	0.51	50.467	0.00	12.53
14	P14	80	2	1360.12	1371.91	1369.38	0.65	50.27	0.68	1360.12	1360.12	0.44	55.36	0.00	47.50	1360.12	1360.12	0.46	50.66	0.00	46.82
15	P15	160	4	2505.42	2565.02	2539.25	8.67	380.53	1.35	2515.02	2505.42	3.06	401.66	0.00	17.20	2515.02	2505.42	2.49	381.43	0.00	17.53
16	P16	160	4	2572.23	2596.83	2580.91	4.16	379.68	0.34	2580.18	2572.23	1.84	394.89	0.00	11.58	2580.18	2572.23	1.82	385.88	0.00	11.60
17	P17	160	4	2709.09	2729.23	2721.28	2.05	384.94	0.45	2709.09	2709.09	0.44	399.21	0.00	30.38	2709.09	2709.09	0.422	389.85	0.00	30.43
18	P18	240	6	3702.85	3807.22	3743.12	11.95	1270.40	1.09	3713.92	3702.85	3.58	1301.32	0.00	23.65	3713.92	3702.85	2.94	1297.40	0.00	23.97
19	P19	240	6	3827.06	3951.21	3946.61	1.29	1275.10	3.12	3839.21	3827.06	3.03	1290.60	0.00	107.55	3839.21	3827.06	3.56	1282.90	0.00	93.54
20	P20	240	6	4058.07	4168.37	4109.06	15.09	1290.30	1.26	4060.37	4058.07	0.53	1257.20	0.00	22.62	4060.37	4058.07	0.48	1297.30	0.00	22.62
21	P21	360	9	5474.84	6101.68	5543.29	145.51	3979.80	1.25	5531.48	5474.84	17.75	4219.40	0.00	12.30	5531.48	5474.84	19.43	3987.30	0.00	12.28
22	P22	360	9	5702.16	5984.87	5736.01	63.39	3991.70	0.59	5741.03	5702.16	15.58	4013.37	0.00	11.81	5741.03	5702.16	11.29	4066.80	0.00	11.98
23	P23	360	9	6095.46	6145.58	6134.91	3.32	4045.70	0.65	6091.43	**6088.96**	0.63	4185.70	−0.11	50.67	6091.43	**6088.96**	0.67	4061.50	−0.11	50.56
24	Pr01	48	4	861.32	883.32	861.32	5.96	12.38	0.00	861.32	861.32	0.52	12.86	0.00	11.63	861.32	861.32	0.48	12.90	0.00	11.64
25	Pr02	96	4	1307.61	1325.54	1319.28	1.69	88.64	0.89	1310.94	**1307.28**	0.84	90.15	−0.03	24.46	1310.94	1307.87	0.84	89.74	0.02	24.46
26	Pr03	144	4	1806.60	1824.32	1813.51	3.08	254.52	0.38	1806.69	1806.60	0.40	290.35	0.00	17.95	1807.13	1806.60	0.60	289.72	0.00	17.32
27	Pr04	192	4	2072.52	2097.33	2087.81	3.14	602.56	0.74	2071.20	**2069.81**	0.48	668.50	−0.13	26.01	2071.20	**2069.95**	0.52	659.91	−0.12	25.96
28	Pr05	240	4	2385.77	2421.44	2403.18	4.84	1210.40	0.73	2393.38	2385.77	1.66	1263.80	0.00	17.34	2396.38	**2385.43**	2.91	1264.40	−0.01	14.03
29	Pr06	288	4	2723.27	2901.13	2813.48	26.29	2035.10	3.31	2697.22	**2687.48**	2.22	2167.00	−1.31	24.44	2691.22	**2687.48**	0.83	2209.73	−1.31	25.24
30	Pr07	72	6	1089.56	1107.82	1089.56	3.89	36.36	0.00	1089.56	1089.56	0.52	39.78	0.00	14.71	1089.56	1089.56	0.48	39.9520	0.00	14.73
31	Pr08	144	6	1666.60	1694.68	1675.99	5.11	256.08	0.56	1666.79	**1665.99**	0.82	287.99	−0.04	17.04	1666.23	**1665.99**	0.52	298.35	−0.04	17.52
32	Pr09	216	6	2153.10	2209.14	2179.53	10.29	901.39	1.23	2151.05	**2134.36**	2.16	929.66	−0.87	17.47	2152.05	**2134.36**	2.11	941.20	−0.87	17.19
33	Pr010	288	6	2921.85	3085.17	3041.39	13.52	2120.30	4.09	2901.95	**2889.59**	3.62	2195.20	−1.10	41.40	2895.95	**2889.59**	1.07	2210.29	−1.10	44.12
**Average execution time**								927.21					1145.72						949.66		

**Note:** the boldface fonts and negative values in each row indicate instances where the proposed algorithms outperform the best results of the Cordeau MDVRP benchmark instances.

### Experimental evaluation of proposed algorithms

Table 3 shows the results of the 33 Cordeau MDVRP benchmark instances, which were ran on the three employed algorithms, namely IWD, IWD-AS, and IWD-ASA. As can be seen in Table 3, the IWD was the least performed method compared to the results of the best known solutions, while the IWD-AS and IWD-ASA were able to find optimal solutions in all the 33 test instances. The superiority of IWD-AS and IWD-ASA are also demonstrated in some cases where their results outperformed the best known solution, indicated in bold and negative signs in the corresponding ‘Best’ and percentage gap ‘Gap’ columns.

Also, to statistically determine the differences among the three algorithms’ solution qualities, two measurement tests were computed, namely the resulting gaps between the three proposed algorithms’ best solution found, and the best known solutions of the other techniques. The second test is the student’s test known as the t-test, which was used to compare the average results of the IWD-SA and IWD-ASA. The gaps were calculated using Eq 20.

$$Gap\left(\%\right)=\frac{Best-BKS}{BKS}\times 100$$ (20)

While the *t* statistics was calculated using the formula given in Eq 21.

$$t=\frac{{\bar{X}}_{1}-{\bar{X}}_{2}}{S_{{\bar{X}}_{1}-{\bar{X}}_{2}}}$$ (21)

$$S_{{\bar{X}}_{1}-{\bar{X}}_{2}}=\sqrt{\frac{\left(n_{1}-1\right)S_{1}^{2}+\left(n_{2}-1\right)S_{2}^{2}}{n_{1}+n_{2}-2}\times \left[\frac{1}{n_{1}}+\frac{1}{n_{2}}\right]}$$ (22)

where

${\overset{-}{X}}_{1}$ = Average value for the set of IWD results

${\overset{-}{X}}_{1}$ = Average value for the other methods results

*S*_1_ = Average value for the other methods results

*S*_1_ = Standard deviation of the other methods including IWD–SA and IWD–ASA

*n*_1_ = frequency of the IWD results for the 10 runs

*n*_2_ = frequencies of the other methods for the 10 runs

The essence of the t-test is to investigate whether there is any significant difference among the average results of the IWD and the other two methods (i.e. IWD-SA and IWD-ASA). In each case the two-tailed P-values obtained were less than 0.0001 and by conventional criteria these differences are considered to be extremely statistically significant. A confidence interval at the 95% confidence level (*t*_0.05_ = 18) was stated. From the results of the t-tests displayed in Table 3, it can be seen that there is statistically significant differences between the average results of the IWD and the other two techniques. In all the test cases, it was obvious that the IWD is outperformed by the IWD-SA and IWD-ASA based on the compared average values of the three techniques. This significant difference in the performance of the other two methods can be attributed to the local search and hill climbing characteristics of the SA introduced into the IWD. These two features of the SA are efficient mechanisms which can assist the IWD to escape being trapped into local minima, and it also increases the explorative and exploitative power of the IWD within the given solution space. One other factor that tends to improve the performance of the hybrid IWD is the efficient calculation of the cost function without compromising the quality of the solution by the SA. This is very important, since the problem cost function is computed at every iteration run of the algorithm. In the current implementation, this bottleneck was avoided by evaluating the cost function based on the difference between the current solution and the neighborhood solution.

**Fig 7:**
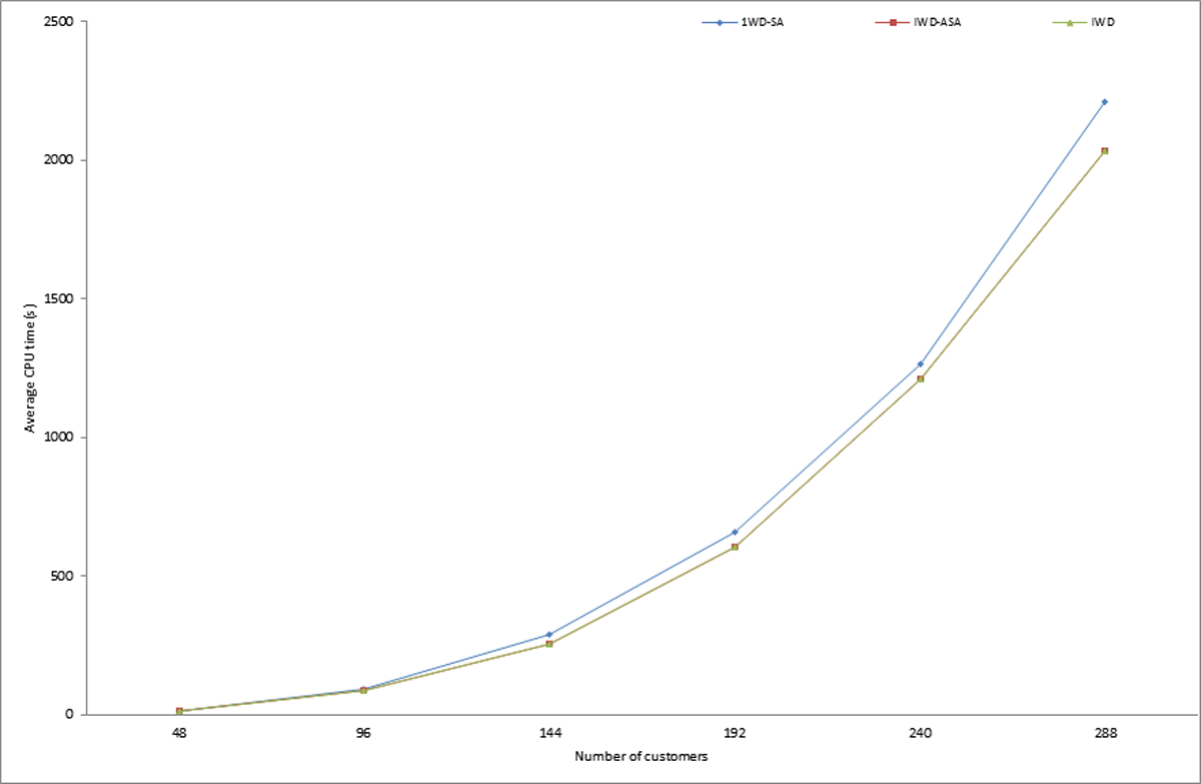
Average running times for IWD, IWD-SA and IWD-ASA on Pr01-Pr06 instances.

**Fig 8:**
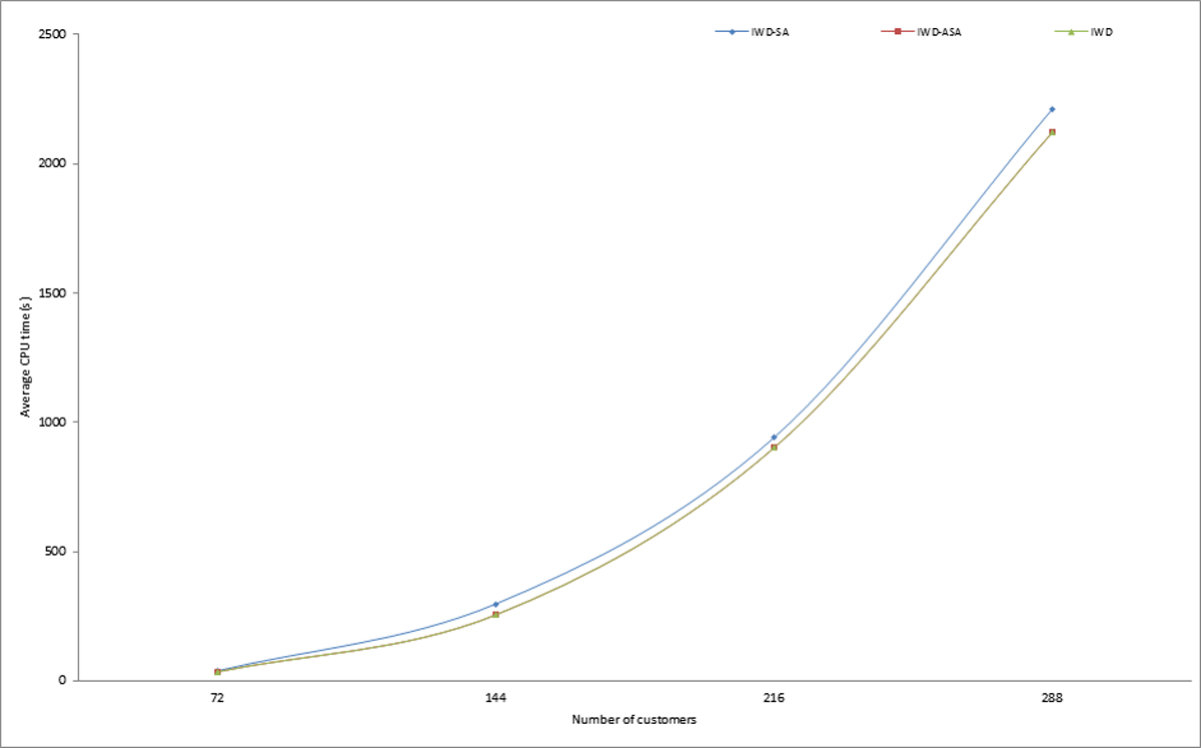
Average running times for IWD, IWD-SA and IWD-ASA on Pr07-Pr10 instances.

**Fig 9:**
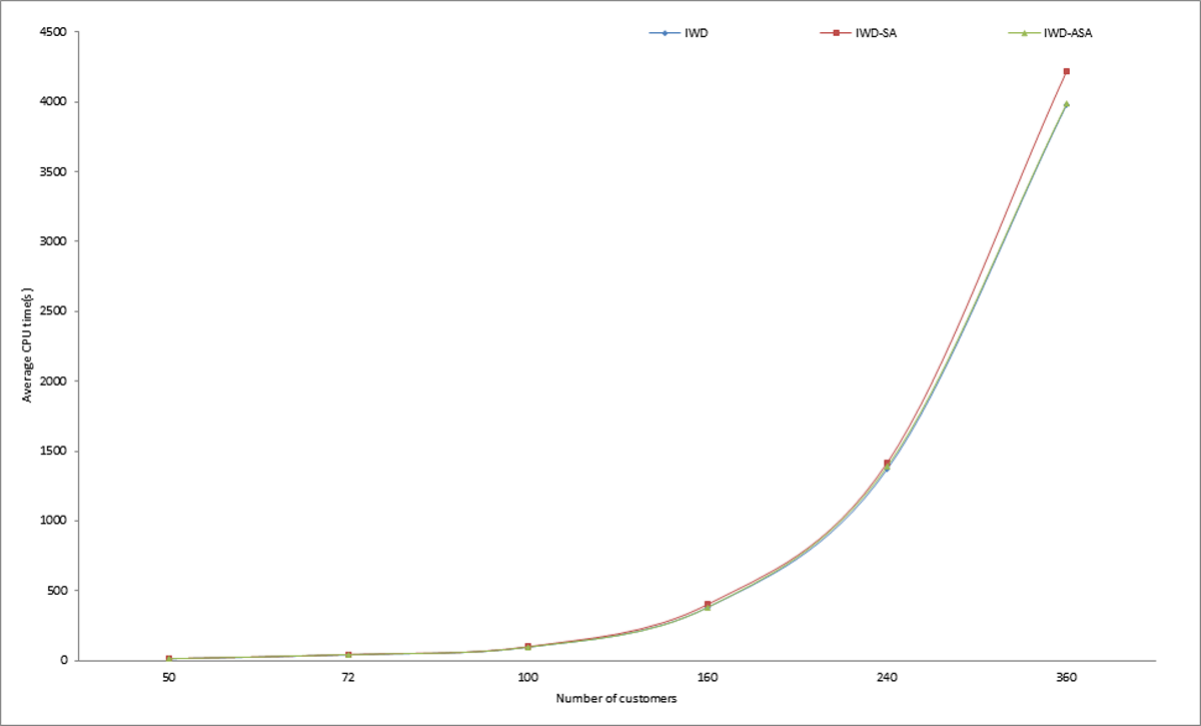
Average running times for IWD, IWD-SA and IWD-ASA on selected instances between P01-P21.

Similarly, in the implementation of the proposed methods, it was observed that the calculation of the exponential probability function alone took approximately one third of the whole algorithm execution time. This however, justifies the initial claim that the computation of the acceptance probability function consumes a copious number of system resources. More specifically, CPU time resulting from the exponential calculation involved in determining the probability of accepting or rejecting a new solution. Similar observations were also pointed out in [54, 55]. Thus, this compelled the researcher to further compare the execution times of the two SA probabilities of acceptance criteria models presented in Eqs 17 and 18. From the experimental results presented in Table 3, it is obvious that IWD-SA took longer CPU time than IWD-ASA. The average execution times of 1,145.72 and 949.66 seconds were recorded for IWD-SA and IWD-ASA respectively, while an average of 927.21 seconds was recorded for the IWD algorithm. The variations in the CPU execution time in seconds for the two methods on some selected problem instances are demonstrated in Figs 7, 8 and 9. However, there were no noticeable significant differences in the solution quality of the two SA techniques aside from the observed wide variation between their processing speeds.

### Experimental evaluation of the algorithms with other methods

**Fig 10:**
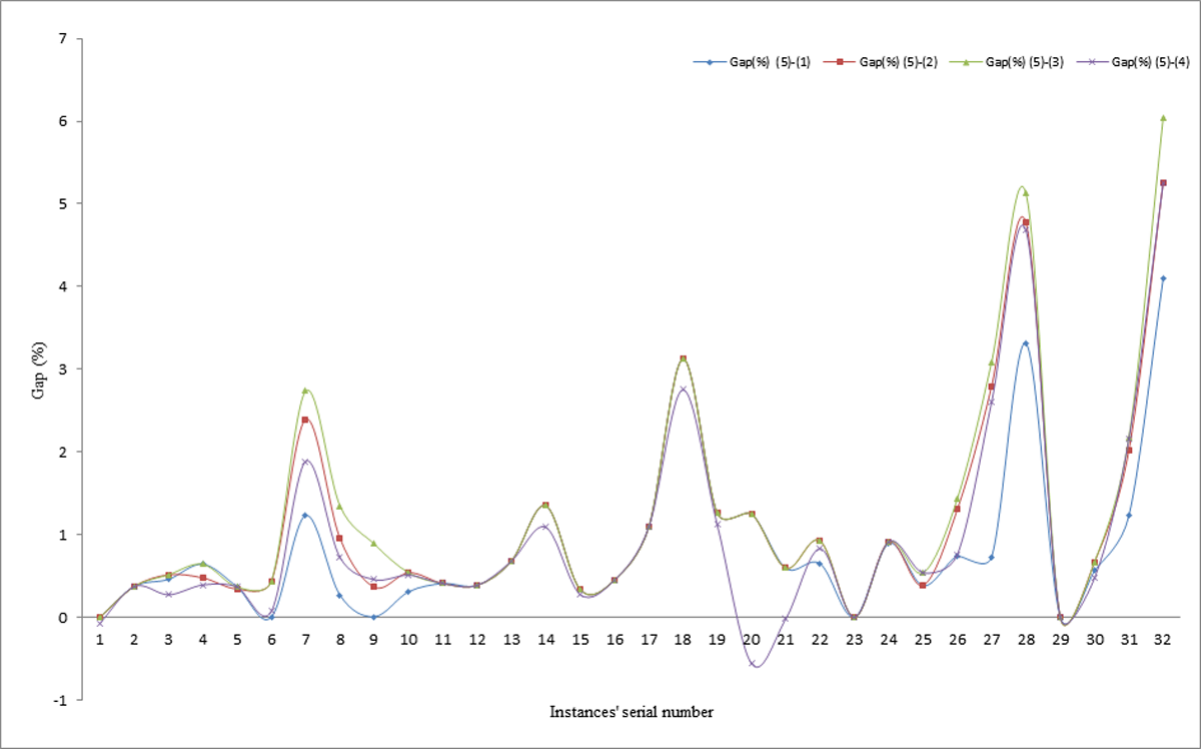
Gaps between IWD and literature techniques.

**Fig 11:**
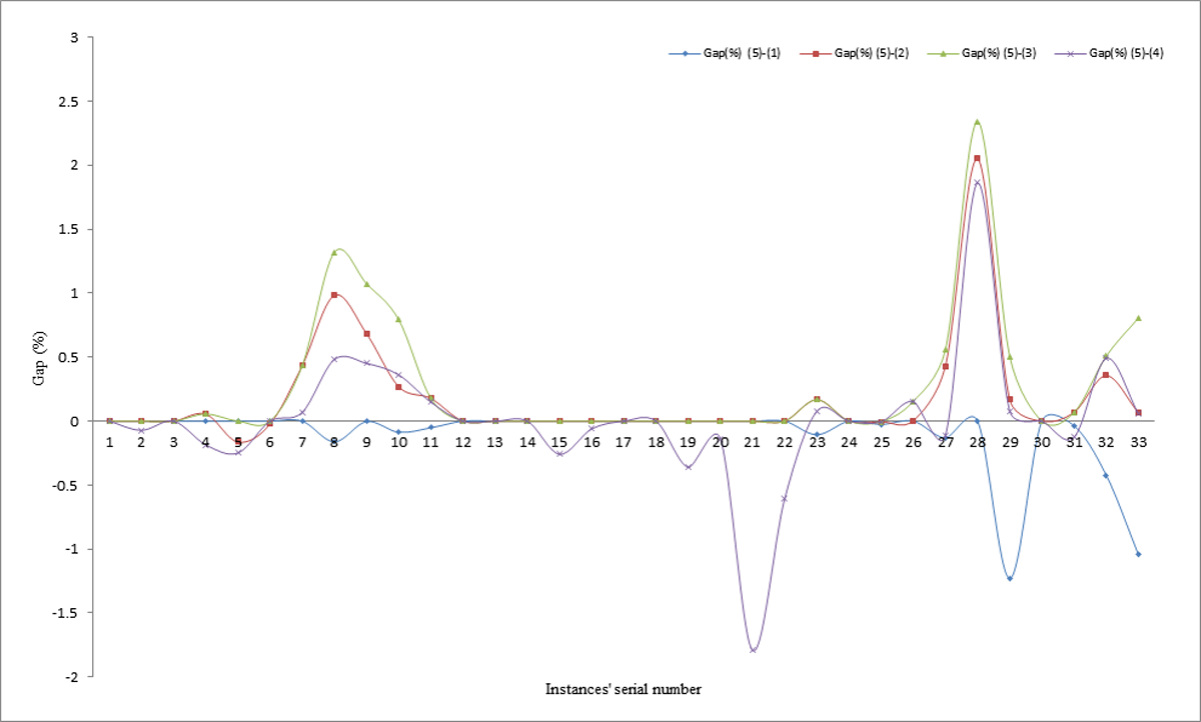
Gaps between IWD-SA and literature techniques.

**Fig 12:**
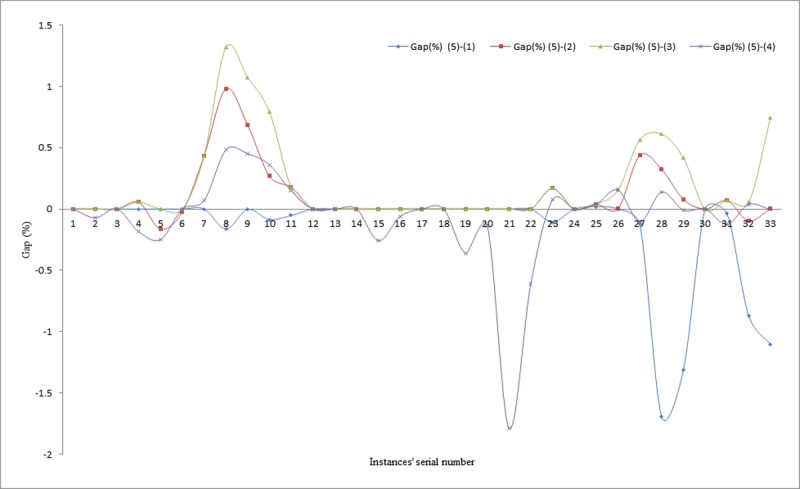
Gaps between IWD-ASA and literature techniques.

This section presents the best solution obtained by each of the proposed methods for comparison with some other approaches available in literature. The gaps in percentages for each of the best results obtained by the different methods are presented for evaluation in Tables 4–6 and similarly, illustrated visually in Figs 10–12. The proposed methods were able to achieve 19 matches out of the 33 existing best known solutions, compared to the results reported in [10], in which 12 matches out of the 33 previous best known solutions were recorded. Similarly, in comparing the average percentage gaps of the three proposed methods, it can be seen that, they are reasonably low, except for the IWD technique. For example, the average gaps between IWD-SA and other techniques are obtained as -0.10% for comparison between IWD-SA and Cordeau *et al*., [37], 0.17% for comparison between IWD-SA and Pisinger and Ropke [39], 0.27% for comparison between IWD-SA and Vidal *et al*. [30], and 0.01% for comparison between IWD-SA and Juan *et al*. [10] for all the 33 instances.

**Table 4:** Comparison of IWD, Cordeau *et al*. [37], Pisinger and Ropke [39], Vidal *et al*. [30], and Juan *et al*. [10].

MDVRP INSTANCE					EXISTING METHODS FROM LITERATURE				PROPOSED METHOD				
INSTANCE					Cordeau *et al*. [37] (1)	Pisinger and Ropke [39] (2)	Vidal *et al*. [30] (3)	Juan et al. [10] (4)	IWD (5)	Gaps between our IWD and other algorithms			
No.	Inst.	n	m	h	Best	Best	Best	Best	Best	Gap(%) (5)-(1)	Gap(%) (5)-(2)	Gap(%) (5)-(3)	Gap(%) (5)-(4)
1	P01	50	4	4	576.87	576.87	576.87	576.87	576.87	0.00	0.00	0.00	0.00
2	P02	50	2	4	473.53	473.53	473.53	473.87	473.53	0.00	0.00	0.00	−0.07
**3**	P03	75	3	5	641.19	641.19	641.19	641.19	643.58	0.37	0.37	0.37	0.37
**4**	P04	100	8	2	1001.59	1001.04	1001.04	1003.45	1006.19	0.46	0.51	0.51	0.27
5	P05	100	5	2	750.03	751.26	750.03	751.90	754.84	0.64	0.48	0.64	0.39
6	P06	100	6	3	876.50	876.70	876.50	876.50	879.71	0.37	0.34	0.37	0.37
7	P07	100	4	4	885.80	881.97	881.97	885.19	885.82	0.00	0.44	0.44	0.07
8	P08	249	14	2	4437.68	4387.38	4372.78	4409.23	4492.39	1.23	2.39	2.74	1.89
9	P09	249	12	3	3900.22	3873.64	3858.66	3882.58	3910.62	0.27	0.95	1.35	0.72
10	P10	249	8	4	3663.02	3650.04	3631.11	3646.67	3663.29	0.00	0.36	0.89	0.46
11	P11	249	6	5	3554.18	3546.06	3546.06	3547.09	3565.17	0.31	0.54	0.54	0.51
12	P12	80	5	2	1318.95	1318.95	1318.95	1318.95	1324.34	0.41	0.41	0.41	0.41
13	P13	80	5	2	1318.95	1318.95	1318.95	1318.95	1324.05	0.39	0.39	0.39	0.39
14	P14	80	5	2	1360.12	1360.12	1360.12	1360.12	1369.38	0.69	0.68	0.68	0.68
15	P15	160	5	4	2505.42	2505.42	2505.42	2511.92	2539.25	1.35	1.35	1.35	1.09
16	P16	160	5	4	2572.23	2572.23	2572.23	2573.78	2580.91	0.34	0.34	0.34	0.28
17	P17	160	5	4	2709.09	2709.09	2709.09	2709.09	2721.28	0.45	0.45	0.45	0.45
18	P18	240	5	6	3702.85	3702.85	3702.85	3702.85	3743.12	1.09	1.09	1.09	1.09
19	P19	240	5	6	3827.06	3827.06	3827.06	3840.91	3946.61	3.12	3.12	3.12	2.75
20	P20	240	5	6	4058.07	4058.07	4058.07	4063.64	4109.06	1.26	1.26	1.26	1.12
21	P21	360	5	9	5474.84	5474.84	5474.84	5574.63	5543.29	1.25	1.25	1.25	−0.56
22	P22	360	5	9	5702.16	5702.16	5702.16	5737.04	5736.01	0.59	0.59	0.59	−0.02
23	P23	360	5	9	6095.46	6078.75	6078.75	6084.32	6134.91	0.64	0.92	0.92	0.83
24	Pr01	48	1	4	861.32	861.32	861.32	861.32	861.32	0.00	0.00	0.00	0.00
25	Pr02	96	2	4	1307.61	1307.34	1307.34	1307.34	1319.28	0.89	0.91	0.91	0.91
26	Pr03	144	3	4	1806.60	1806.53	1803.80	1803.80	1813.51	0.38	0.39	0.54	0.54
27	Pr04	192	4	4	2072.52	2060.93	2058.31	2072.10	2087.81	0.74	1.30	1.43	0.76
28	Pr05	240	5	4	2385.77	2337.84	2331.20	2342.20	2403.18	0.73	2.79	3.09	2.61
29	Pr06	288	6	4	2723.27	2685.35	2676.30	2687.73	2813.48	3.31	4.77	5.13	4.68
30	Pr07	72	1	6	1089.56	1089.56	1089.56	1089.56	1089.56	0.00	0.00	0.00	0.00
31	Pr08	144	2	6	1666.60	1664.85	1664.85	1668.08	1675.99	0.56	0.67	0.67	0.47
32	Pr09	216	3	6	2153.10	2136.42	2133.20	2133.56	2179.53	1.23	2.02	2.17	2.15
33	Pr010	288	4	6	2921.85	2889.49	2868.26	2889.70	3041.39	4.09	5.26	6.04	5.25
	Average gaps									0.82%	1.10%	1.20%	0.93%

**Table 5:** Comparison of IWD-SA, Cordeau *et al*. [37], Pisinger and Ropke [39], Vidal *et al*. [30], and Juan *et al*. [10].

MDVRP INSTANCE					EXISTING METHODS FROM LITERATURE				PROPOSED METHOD				
INSTANCE					Cordeau *et al*. [37] (1)	Pisinger and Ropke [39] (2)	Vidal *et al*. [30] (3)	Juan et al. [10] (4)	IWD-SA (5)	Gaps between our IWD and other algorithms			
No.	Inst.	n	m	h	Best	Best	Best	Best	Best	Gap(%) (5)-(1)	Gap(%) (5)-(2)	Gap(%) (5)-(3)	Gap(%) (5)-(4)
1	P01	50	4	4	576.87	576.87	576.87	576.87	576.87	0.00	0.00	0.00	0.00
2	P02	50	2	4	473.53	473.53	473.53	473.87	473.53	0.00	0.00	0.00	−0.07
3	P03	75	3	5	641.19	641.19	641.19	641.19	641.19	0.00	0.00	0.00	0.00
4	P04	100	8	2	1001.59	1001.04	1001.04	1003.45	1001.59	0.00	0.05	0.05	−1.89
5	P05	100	5	2	750.03	751.26	750.03	751.90	750.03	0.00	−0.16	0.00	−0.25
6	P06	100	6	3	876.50	876.70	876.50	876.50	876.50	0.00	−0.02	0.00	0.00
7	P07	100	4	4	885.80	881.97	881.97	885.19	885.80	0.00	0.43	0.43	0.07
8	P08	249	14	2	4437.68	4387.38	4372.78	4409.23	4430.48	−0.16	0.98	1.32	0.48
9	P09	249	12	3	3900.22	3873.64	3858.66	3882.58	3900.10	0.00	0.68	1.07	0.45
10	P10	249	8	4	3663.02	3650.04	3631.11	3646.67	3659.84	−0.09	0.27	0.79	0.36
11	P11	249	6	5	3554.18	3546.06	3546.06	3547.09	3552.34	−0.05	0.18	0.18	0.15
12	P12	80	5	2	1318.95	1318.95	1318.95	1318.95	1318.95	0.00	0.00	0.00	0.00
13	P13	80	5	2	1318.95	1318.95	1318.95	1318.95	1318.95	0.00	0.00	0.00	0.00
14	P14	80	5	2	1360.12	1360.12	1360.12	1360.12	1360.12	0.00	0.00	0.00	0.00
15	P15	160	5	4	2505.42	2505.42	2505.42	2511.92	2505.42	0.00	0.00	0.00	−0.26
16	P16	160	5	4	2572.23	2572.23	2572.23	2573.78	2572.23	0.00	0.00	0.00	−0.06
17	P17	160	5	4	2709.09	2709.09	2709.09	2709.09	2709.09	0.00	0.00	0.00	0.00
18	P18	240	5	6	3702.85	3702.85	3702.85	3702.85	3702.85	0.00	0.00	0.00	0.00
19	P19	240	5	6	3827.06	3827.06	3827.06	3840.91	3827.06	0.00	0.00	0.00	−0.36
20	P20	240	5	6	4058.07	4058.07	4058.07	4063.64	4058.07	0.00	0.00	0.00	−0.14
21	P21	360	5	9	5474.84	5474.84	5474.84	5574.63	5474.84	0.00	0.00	0.00	−1.79
22	P22	360	5	9	5702.16	5702.16	5702.16	5737.04	5702.16	0.00	0.00	0.00	−0.61
23	P23	360	5	9	6095.46	6078.75	6078.75	6084.32	6088.96	−0.11	0.17	0.17	0.08
24	Pr01	48	1	4	861.32	861.32	861.32	861.32	861.32	0.00	0.00	0.00	0.00
25	Pr02	96	2	4	1307.61	1307.34	1307.34	1307.34	1307.28	−0.03	0.00	0.00	0.00
26	Pr03	144	3	4	1806.6	1806.53	1803.80	1803.80	1806.60	0.00	0.00	0.16	0.16
27	Pr04	192	4	4	2072.52	2060.93	2058.31	2072.10	2069.81	−0.13	0.43	0.56	−0.11
28	Pr05	240	5	4	2385.77	2337.84	2331.20	2342.20	2345.77	−1.68	0.34	0.63	0.15
29	Pr06	288	6	4	2723.27	2685.35	2676.30	2687.73	2687.48	−1.31	0.08	0.42	−0.01
30	Pr07	72	1	6	1089.56	1089.56	1089.56	1089.56	1089.56	0.00	0.00	0.00	0.00
31	Pr08	144	2	6	1666.60	1664.85	1664.85	1668.08	1665.99	−0.04	0.07	0.07	−0.13
32	Pr09	216	3	6	2153.10	2136.42	2133.20	2133.56	2134.36	−0.87	−0.10	0.05	0.04
33	Pr010	288	4	6	2921.85	2889.49	2868.26	2889.70	2889.59	−1.10	0.00	0.74	0.00
	Average gaps									−0.10%	0.17%	0.27%	0.01%

**Table 6:** Comparison of IWD -ASA, Cordeau *et al*. [37], Pisinger and Ropke [39], Vidal *et al*. [30], and Juan *et al*. [10].

MDVRP INSTANCE					EXISTING METHODS FROM LITERATURE				PROPOSED METHOD				
INSTANCE					Cordeau et al. [37] (1)	Pisinger and Ropke [39] (2)	Vidal *et al*. [30] (3)	Juan et al. [10] (4)	IWD-ASA (5)	Gaps between our IWD-ASA and other algorithms			
No.	Inst.	n	m	h	Best	Best	Best	Best	Best	Gap(%) (5)-(1)	Gap(%) (5)-(2)	Gap(%) (5)-(3)	Gap(%) (5)-(4)
1	P01	50	4	4	576.87	576.87	576.87	576.87	576.87	0.00	0.00	0.00	0.00
2	P02	50	2	4	473.53	473.53	473.53	473.87	473.53	0.00	0.00	0.00	−0.07
3	P03	75	3	5	641.19	641.19	641.19	641.19	641.19	0.00	0.00	0.00	0.00
4	P04	100	8	2	1001.59	1001.04	1001.04	1003.45	1001.59	0.00	0.05	0.05	−0.19
5	P05	100	5	2	750.03	751.26	750.03	751.90	750.03	0.00	−0.16	0.00	−0.25
6	P06	100	6	3	876.50	876.70	876.50	876.50	876.50	0.00	−0.02	0.00	0.00
7	P07	100	4	4	885.80	881.97	881.97	885.19	885.80	0.00	0.43	0.43	0.07
8	P08	249	14	2	4437.68	4387.38	4372.78	4409.23	4430.48	−0.16	0.98	1.32	0.48
9	P09	249	12	3	3900.22	3873.64	3858.66	3882.58	3900.10	0.00	0.68	1.07	0.45
10	P10	249	8	4	3663.02	3650.04	3631.11	3646.67	3659.84	−0.09	0.27	0.79	0.36
11	P11	249	6	5	3554.18	3546.06	3546.06	3547.09	3552.34	−0.05	0.18	0.178	0.15
12	P12	80	5	2	1318.95	1318.95	1318.95	1318.95	1318.95	0.00	0.00	0.00	0.00
13	P13	80	5	2	1318.95	1318.95	1318.95	1318.95	1318.95	0.00	0.00	0.00	0.00
14	P14	80	5	2	1360.12	1360.12	1360.12	1360.12	1360.12	0.00	0.00	0.00	0.00
15	P15	160	5	4	2505.42	2505.42	2505.42	2511.92	2505.42	0.00	0.00	0.00	−0.26
16	P16	160	5	4	2572.23	2572.23	2572.23	2573.78	2572.23	0.00	0.00	0.00	−0.06
17	P17	160	5	4	2709.09	2709.09	2709.09	2709.09	2709.09	0.00	0.00	0.00	0.00
18	P18	240	5	6	3702.85	3702.85	3702.85	3702.85	3702.85	0.00	0.00	0.00	0.00
19	P19	240	5	6	3827.06	3827.06	3827.06	3840.91	3827.06	0.00	0.00	0.00	−0.36
20	P20	240	5	6	4058.07	4058.07	4058.07	4063.64	4058.07	0.00	0.00	0.00	−0.14
21	P21	360	5	9	5474.84	5474.84	5474.84	5574.63	5474.84	0.00	0.00	0.00	−1.79
22	P22	360	5	9	5702.16	5702.16	5702.16	5737.04	5702.16	0.00	0.00	0.00	−0.61
23	P23	360	5	9	6095.46	6078.75	6078.75	6084.32	6088.96	−0.11	0.17	0.17	0.08
24	Pr01	48	1	4	861.32	861.32	861.32	861.32	861.32	0.00	0.00	0.00	0.00
25	Pr02	96	2	4	1307.61	1307.34	1307.34	1307.34	1307.87	0.02	0.04	0.04	0.04
26	Pr03	144	3	4	1806.60	1806.53	1803.80	1803.80	1806.60	0.00	0.00	0.16	0.16
27	Pr04	192	4	4	2072.52	2060.93	2058.31	2072.10	2069.95	−0.12	0.44	0.57	−0.10
28	Pr05	240	5	4	2385.77	2337.84	2331.20	2342.20	2345.43	−1.69	0.32	0.61	0.14
29	Pr06	288	6	4	2723.27	2685.35	2676.30	2687.73	2687.48	−1.31	0.08	0.42	−0.01
30	Pr07	72	1	6	1089.56	1089.56	1089.56	1089.56	1089.56	0.00	0.00	0.00	0.00
31	Pr08	144	2	6	1666.60	1664.85	1664.85	1668.08	1665.99	−0.04	0.07	0.07	−0.13
32	Pr09	216	3	6	2153.10	2136.42	2133.20	2133.56	2134.36	−0.87	−0.01	0.05	0.05
33	Pr010	288	4	6	2921.85	2889.49	2868.26	2889.70	2889.59	−1.10	0.00	0.74	0.00
	Average gaps									−0.17%	0.10%	0.20%	−0.06%

### Statistical and convergence behavior analysis

In addition to the previous comparison made, two statistical tests were carried out, using the results presented in Tables 4, 5 and 6 in order to obtain more rigorous and fair conclusions regarding the performance and convergence behavior of each of the compared algorithms. Firstly, Friedman’s non-parametric test was conducted on the five different algorithms and the result is presented in Table 7. Secondly, an application was conducted of post hoc analysis with Wilcoxon signed-rank tests using IWD, IWD-SA, and IWD-ASA as controlled algorithms the result of which is presented in Table 8.

**Table 7:** Average ranking returned by Friedman’s non-parametric test for the 33 MDVRP instances.

Ranks					
Algorithm	Mean Rank	Algorithm	Mean Rank	Algorithm	Mean Rank
Cordeau *et al*. [37]	3.03	Cordeau *et al*. [37]	3.75	Cordeau *et al*. [37]	3.70
Pisinger and Ropke [39]	2.30	Pisinger and Ropke [39]	2.72	Pisinger and Ropke [39]	2.73
Vidal *et al*. [30]	1.85	Vidal *et al*. [30]	2.19	Vidal *et al*. [30]	2.18
Juan *et al*. [10]	3.14	Juan *et al*. [10]	3.69	Juan *et al*. [10]	3.67
IWD	4.68	IWD-SA	2.66	IWD-ASA	2.73

**Table 8:** Application of post hoc analysis with Wilcoxon signed-rank tests using IWD, IWD-SA, and IWD-ASA as controlled algorithms.

		Test Statistics^b^		
	IWD–Cordeau *et al*. [37]	IWD–Pisinger and Ropke [39]	IWD–Vidal *et al*. [30]	IWD–Juan *et al*. [10]
Z	−4.703^a^	−4.703^a^	−4.703^a^	−4.268^a^
Asymp. Sig. (2-tailed)	.000	.000	.000	.000
	IWD_SA—Cordeau *et al*. [37]	IWD_SA—Pisinger and Ropke [39]	IWD_SA—Vidal *et al*. [30]	IWD_SA—Juan *et al*. [10]
Z	−3.621^a^	−1.288^a^	−2.343^a^	−1.251^a^
Asymp. Sig. (2-tailed)	.000	.198	.019	.211
	IWD_ASA—Cordeau *et al*. [37]	IWD_ASA—Pisinger and Ropke [39]	IWD_ASA—Vidal *et al*. [30]	IWD_ASA—Juan *et al*. [10]
Z	−3.527^a^	−1.449^a^	−2.533^a^	−1.090^a^
Asymp. Sig. (2-tailed)	.000	.147	.011	.276

a. Based on positive ranks.

b. Wilcoxon Signed Ranks Test

Friedman’s non-parametric test for multiple comparisons conducted indicated that there was a statistically significant difference among the results of the five (5) algorithms. The test was conducted by taking into account the confidence interval which was stated at the 95% confidence level. As shown in Table 7, Friedman’s test revealed that there were statistically significant differences among the techniques whilst running, *X*^*2*^(4) = 82.68, *p*-value = 0.000 for the comparison between IWD and other methods; *X*^*2*^(4) = 37.60, *p*-value = 0.000 for the comparison between IWD-SA and other methods; and *X*^*2*^(4) = 37.52, *p*-value = 0.000 for the comparison between IWD-ASA and other methods. Since the Friedman test result was statistically significant, there was a need to still run the post hoc tests, so that it could be ascertained exactly which of the algorithms performed better.

To properly evaluate the statistical significance of the efficient performance of the proposed IWD, IWD-SA and IWD-ASA algorithms, the post hoc tests have been conducted further by using each of the three methods as the controlled algorithm in all the comparisons made. However, the post hoc analysis with Wilcoxon signed-rank tests conducted with a Bonferroni correction applied, resulted in a new significance level set at *p* < 0.0125 (i.e. 0.05/4, since 4 comparisons were made). Analyzing the test statistics data shown in Table 8, it can be seen that at *p* < 0.0125 significance level, the proposed methods produced results that were significantly better than Cordeau *et al*. [37] and Vidal *et al*. [30]. However, there were no statistically significant differences between the results obtained by the two proposed methods (that is, IWD-SA and IWD-ASA) (see Pisinger and Ropke [39], and Juan *et al*. [10]), since their resulting p-values were greater than the computed p-value of 0.0125. Therefore, it can be concluded that the two proposed methods are statistically significantly better than the IWD and Cordeau *et al*. [37] for solving MDVRP problems at 95% confidence level.

**Fig 13:**
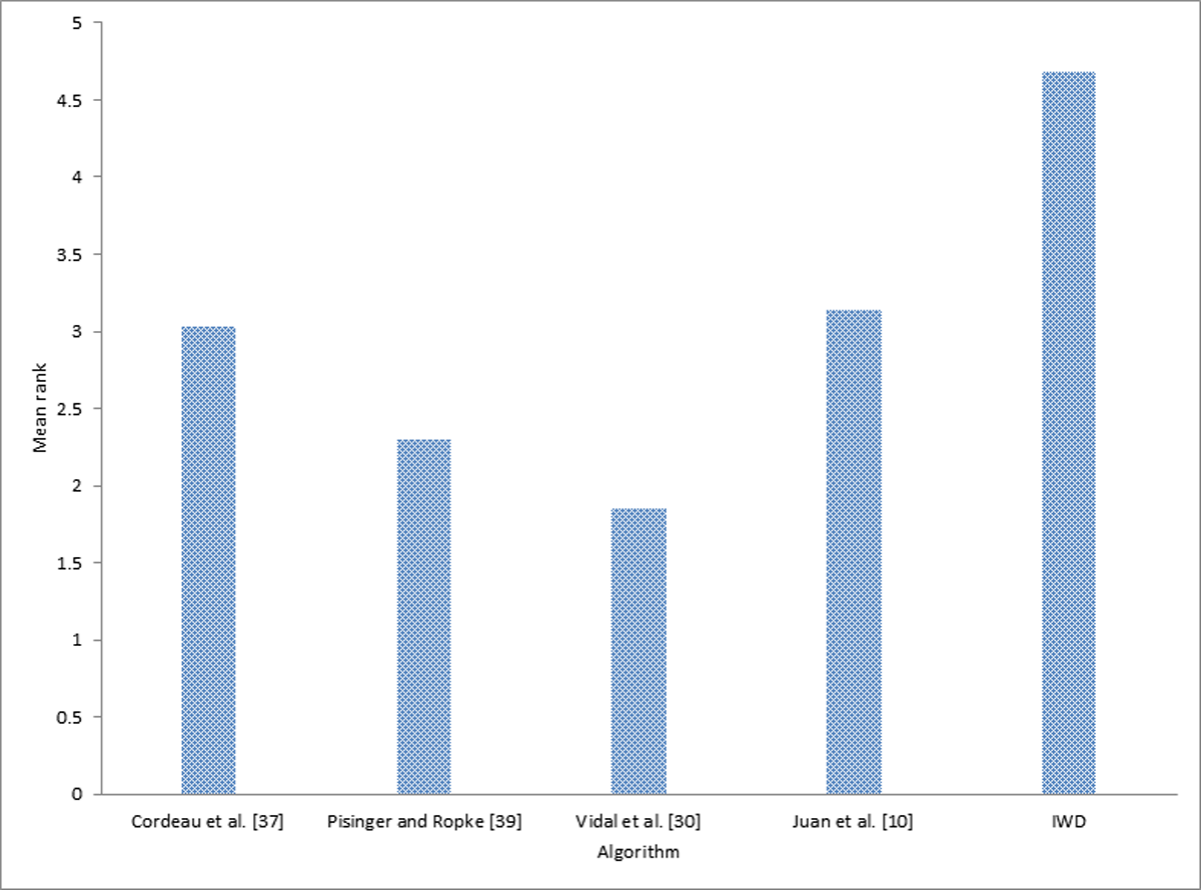
Mean rank of the IWD algorithm with other methods.

**Fig 14:**
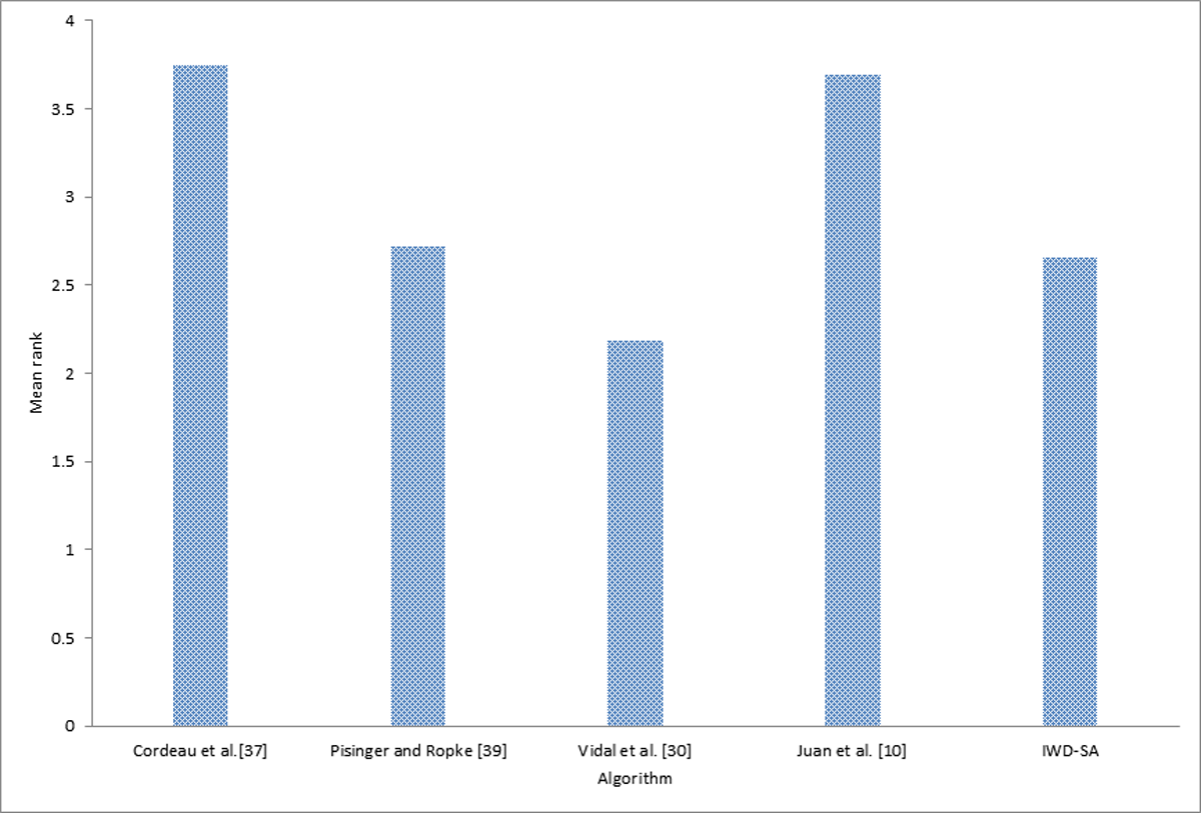
Mean rank of the IWD-SA algorithm with other methods.

**Fig 15:**
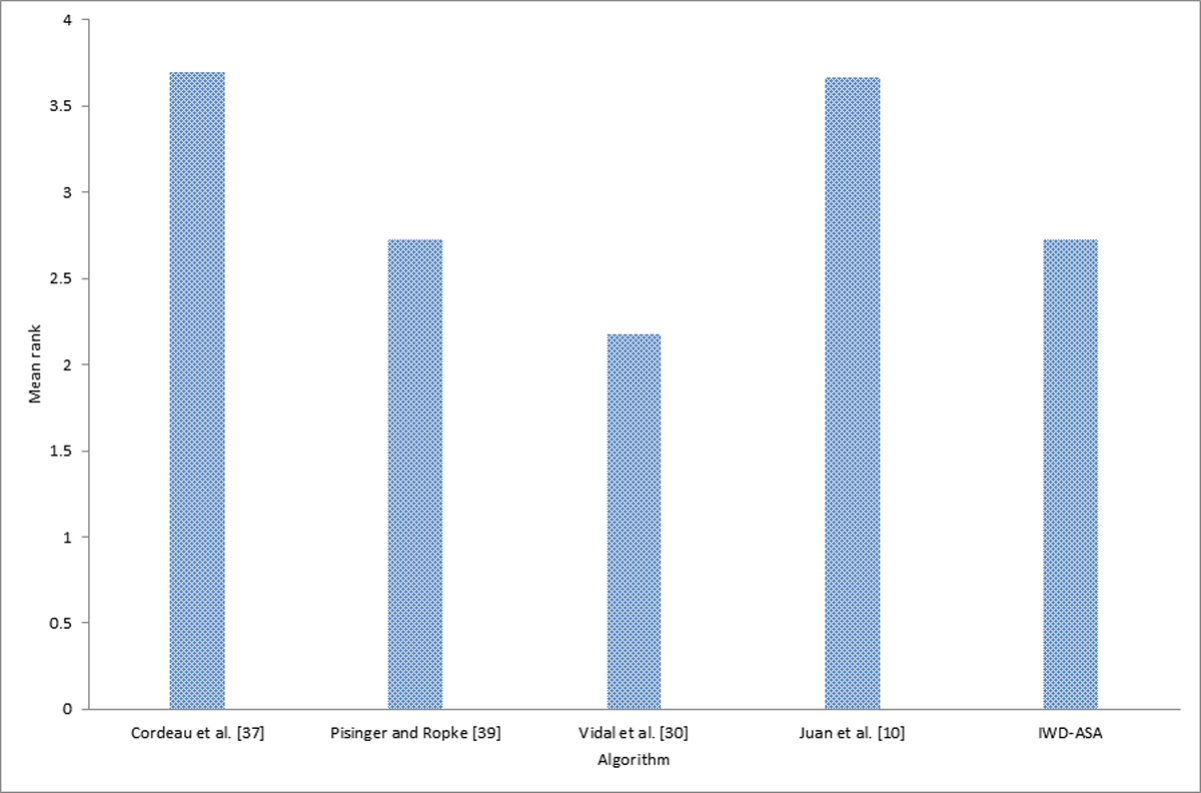
Mean rank of the IWD-ASA algorithm with other methods.

Table 7 reports the average ranks returned by Friedman’s non-parametric test for the five set of algorithm on 33 MDVRP instances. As shown in Table 7, Vidal *et al*. [30] yielded the best performance among all the other methods, while two of the current methods namely, IWD-SA and IWD-ASA together with Pisinger and Ropke [39] competed equally for the second position in the analysis. However, the IWD performed poorly in either case. (Figs 13, 14 and 15 graphically depict this ranking evaluation).

**Fig 16:**
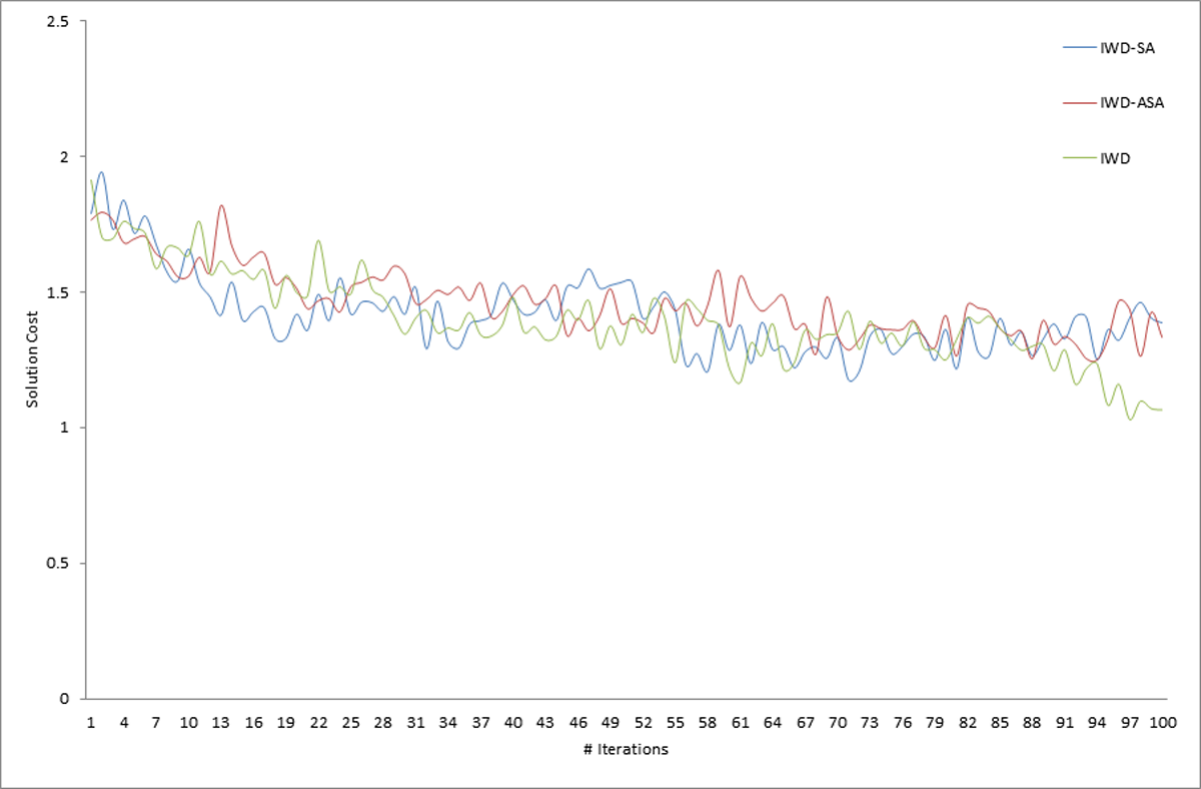
Convergence trends of IWD, IWD-SA, and IWD-ASA for the P01 instance.

**Fig 17:**
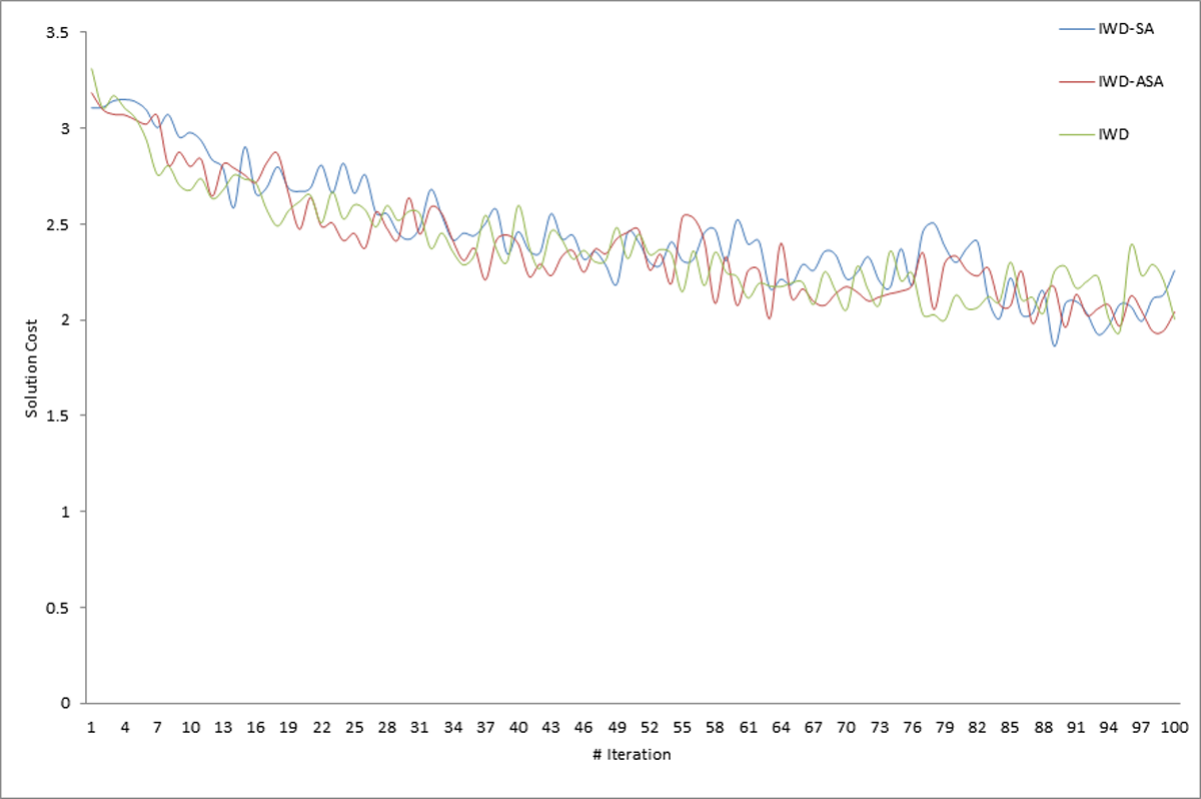
Convergence trends of IWD, IWD-SA, and IWD-ASA for the P04 instance.

Evaluating the different methods proposed in this paper, is based on each algorithm’s solution qualities and speed of convergence, as shown in Figs 16 and 17. These two figures depict that both the IWD-SA and IWD-ASA competed favorably well in terms of solution quality and speed of convergence compared to the standard IWD, despite the additional computational cost incurred by the IWD-SA. This further support the significant contributions of the different strategies employed to implement the proposed algorithms. Some of these main strategies include the use of efficient cost function, approximation of SA’s probability of acceptance criteria and the use of SA as an alternative local search technique to intensify the entire search process, and at the same time to accelerate the convergence speed of the proposed hybrid algorithms.

In summarizing the whole analysis, from the computational result obtained in Table 3, it is concluded that there is a significant difference in the CPU execution time between IWD-SA and IWD-ASA, with the approximate version of the SA having the least execution time. This reduction in the algorithms’ run time indirectly resulted in accelerating the convergence speed of the IWD-SA without compromising its solution quality. Therefore, it can be stated that the approximate method of the hybrid algorithm (IWD-ASA) performed better than its IWD-SA counterpart in terms of CPU time. After analyzing the results presented in Tables 4, 5, and 6, it is concluded that the two proposed hybrid algorithms achieved high quality solutions compared to other methods described in the literature (19 out of 33). Specifically, it is also concluded that the two hybrid methods performed well in MDVRP instances with up to 360 nodes (customers). In summary, as shown in all the results evaluated, the proposed methods can be employed as alternative hybrid metaheuristics for solving other similar MDVRP problems.

## Conclusion and future research

In this paper the multi-depot vehicle routing problem has been studied. Presented are two hybrid metaheuristics that incorporate both the exponential and approximate version of the simulated annealing into the basic intelligent water drops algorithm. This study utilized these two methods of the SA solution to develop a better alternative algorithm for the MDVRP NP-hard problem. The proposed algorithms (IWD-SA and IWD-ASA) were tested on two sets of Cordeau’s MDVRP benchmark instances, covering instances P01-P23 and Pr01-Pr10. Two different statistical analyses were also conducted to verify the performances of the proposed methods against some existing techniques in the literature. The obtained results show that the two methods are effective and efficient in solving MDVRP within a reasonable amount of computational effort and high solution qualities.

A statistical analysis was also conducted to compare the performances of the proposed methods. Results of the analysis revealed that there are no significant statistical differences in terms of solution qualities obtained by the two SA methods. However, statistical results also revealed that the exponential implementation of the SA consumed more CPU time than the approximate method. For example, the IWD-SA took an average of 1,145.72 seconds to run all the 33 benchmark instances considered in the implementation, in comparison with 949.66 and 927.21 seconds for IWD-ASA and IWD. The basic IWD was outperformed by the two IWS-SA and IWD-ASA hybrid methods, which is a good indication that SA actually contributed to improving the solution quality of the standard IWD algorithm. Comparisons with other methods show that there are significant statistical differences among the compared algorithms from the literature with the proposed IWD-SA based methods, while the classical IWD was outperformed by all the other algorithms using the same parameter settings with the SA variants. Therefore, these results indicate that both IWD-SA and IWD-ASA are more effective alternative candidate algorithms than the compared methods, for solving multi-depot vehicle routing problem.

As a direction for further research, the proposed methods could be further improved and subsequently applied to solve other variants of capacitated vehicle routing problems. Also a better approach that could be exploited is to build a look-up table for a set of values over the range Δ*f*/*t* to be used as alternative to the exponential calculation of the probability of acceptance criteria.
